# Footwear and insole design features for offloading the diabetic at risk foot—A systematic review and meta‐analyses

**DOI:** 10.1002/edm2.132

**Published:** 2020-04-11

**Authors:** Richard Collings, Jennifer Freeman, Jos M. Latour, Joanne Paton

**Affiliations:** ^1^ School of Health Professions Faculty of Health: Medicine, Dentistry and Human Sciences University of Plymouth Plymouth UK; ^2^ Department of Podiatry, Torbay and South Devon NHS Foundation Trust Plymouth UK; ^3^ School of Nursing and Midwifery Faculty of Health: Medicine, Dentistry and Human Sciences University of Plymouth Plymouth UK

**Keywords:** diabetic foot, footwear, insoles, offloading, prevention, systematic review

## Abstract

The aim of this systematic review was to identify the best footwear and insole design features for offloading the plantar surface of the foot to prevent foot ulceration in people with diabetic peripheral neuropathy. We searched multiple databases for published and unpublished studies reporting offloading footwear and insoles for people with diabetic neuropathy and nonulcerated feet. Primary outcome was foot ulcer incidence; other outcome measures considered were any standardized kinetic or kinematic measure indicating loading or offloading the plantar foot. Fifty‐four studies, including randomized controlled studies, cohort studies, case‐series, and a case‐controlled and cross‐sectional study were included. Three meta‐analyses were conducted and random‐effects modelling found peak plantar pressure reduction of arch profile (37 kPa (MD, −37.5; 95% CI, −72.29 to −3.61; *P* < .03), metatarsal addition (35.96 kPa (MD, −35.96; 95% CI, −57.33 to −14.60; *P* < .001) and pressure informed design 75.4 kPa (MD, −75.4 kPa; 95% CI, −127.4 to −23.44 kPa; *P* < .004).The remaining data were presented in a narrative form due to heterogeneity. This review highlights the difficulty in differentiating the effect of different insole and footwear features in offloading the neuropathic diabetic foot. However, arch profiles, metatarsal additions and apertures are effective in reducing plantar pressure. The use of pressure analysis to enhance the effectiveness of the design of footwear and insoles, particularly through modification, is recommended.

## Graphical abstract

The aim of this systematic review was to identify the best footwear and insole design features for offloading the plantar surface of the foot to prevent foot ulceration in people with diabetic peripheral neuropathy. Fifty‐four studies were included. Three meta‐analyses were conducted and random‐effects modelling found peak plantar pressure reduction of arch profile, metatarsal addition and pressure‐informed design.

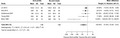

## 1 INTRODUCTION

Foot ulceration is amongst the most serious complications of diabetes mellitus.^1^ It is expected that 19%‐34% of people with diabetes will develop a foot ulcer at some point.^2^ Foot ulceration is known to precede 80% of all diabetic lower limb amputations.^3^, ^4^ A longitudinal study of a diabetic community reported new ulcer incidence as an estimated 2% annually^5^ whilst other studies have noted ulcer reoccurrence rates of 30%‐40% in the first year after an ulcer episode.^2^, ^6^, ^7^ Prevention of foot ulceration occurrence and reoccurrence are now recognized as key strategies in reducing the concomitant burden to patients with diabetes and the healthcare system.^8^

The cause of diabetic foot ulceration is multifactorial.^9^ However, reducing high plantar loads or foot pressures is one mechanism by which foot ulceration may be prevented.^10^ Elevated dynamic plantar pressures during locomotion contribute to the development of plantar diabetic foot ulcers when in the presence of neuropathy.^11^, ^12^ Guidelines recommended that people with diabetes wear appropriate ‘diabetic footwear’ designed to reduce repetitive stresses at all times.^13^ Systematic reviews have demonstrated the effectiveness of footwear and insoles in offloading the plantar load under the foot and preventing ulceration.^14^, ^15^, ^16^, ^17^, ^18^ However, these have not identified the best insole design or feature and footwear specification or modification for use when reducing plantar load for foot ulcer prevention in people with diabetes and neuropathy.

Therefore, the purpose of this systematic literature review was to identify the best footwear and insole design features for offloading the plantar surface of the foot to prevent foot ulceration in people with diabetes. It is anticipated that this information will inform a standardized protocol for the clinical design of therapeutic insoles and footwear to offload the foot and reduce ulcer risk in people with diabetes and neuropathy.

More specifically, the objectives are to identify the key design features with regard to the following:

- profile/shape of the insole, shoe upper and shoe outsole
- material type and properties of the insole and shoe outsole
- modifications made to the insole and shoe outsole
- fabrication techniques used for the insole and shoe

## 2 METHODS

This systematic review was performed and reported according to the Preferred Reporting Items for Systematic Reviews and Meta‐Analyses (PRISMA) Guidance.^19^ The systematic review was prospectively registered on the PROSPERO database for systematic reviews (CRD42017072816).

The population of interest was adults over 18 years of age with type 1 or 2 diabetes mellitus and peripheral neuropathy. The primary outcome was foot ulcer incidence; other outcome measures considered were any standardized kinetic or kinematic measure indicating loading or offloading the plantar foot (such as plantar pressure, pressure‐time integral, total contact area, dynamic measures of centre of pressure trajectory or velocity) and any standardized clinical measure indicating loading/offloading of the plantar foot (such as callus/lesion reduction). Side effects/adverse events as a result of the design features were additional outcomes of interest. We excluded studies on people with active ulceration, major amputation of the foot or Charcot arthropathy because we considered that the unique pathomechanics and gross deformity associated with the severity of these conditions would unduly influence the design features of the footwear and insoles.

This review included both experimental and epidemiological study designs including randomized controlled trials, non–randomized controlled trials, quasi‐experimental, before and after studies, prospective and retrospective cohort studies and analytical cross‐sectional studies. Studies were included if they made one of the following comparisons: footwear and/or insole design feature compared with another therapeutic footwear and/or insole design feature; footwear and/or insole design feature compared with no intervention. Qualitative studies, case reports and systematic reviews were excluded.

The initial literature search was performed on 27 July 2016 by one researcher (RC) and covered publications in English and was not restricted by date. The search was updated on 27 December 2017 and 30 October 2019. The following databases were searched: Excerpta Medica Database (EMBASE) via Ovid, Medline and Cochrane Database of Systematic Reviews, AMED (EBSCO), Cumulative Index to Nursing and Allied Health Literature (CINAHL), MEDLINE, Joanna Briggs Institute Database of Systematic Reviews and PROSPERO. A search for unpublished studies was undertaken in EThOS, Pearl, Web of Science, Google Scholar and SIGLE. The search strings were prepared with the help of an evidence synthesis specialist. An example of the search from one of the databases is provided in Appendix S1. Title and abstract of all papers retrieved by the literature search were screened independently by two researchers (RC and JP) to determine whether the paper met the inclusion criteria with disagreements resolved by discussion. Full‐text articles were then retrieved and further screened by two researchers (RC and JP) independently for inclusion in the review. In addition, a hand search was undertaken using the references from journal articles.

## 3 RESULTS

**Figure 1:**
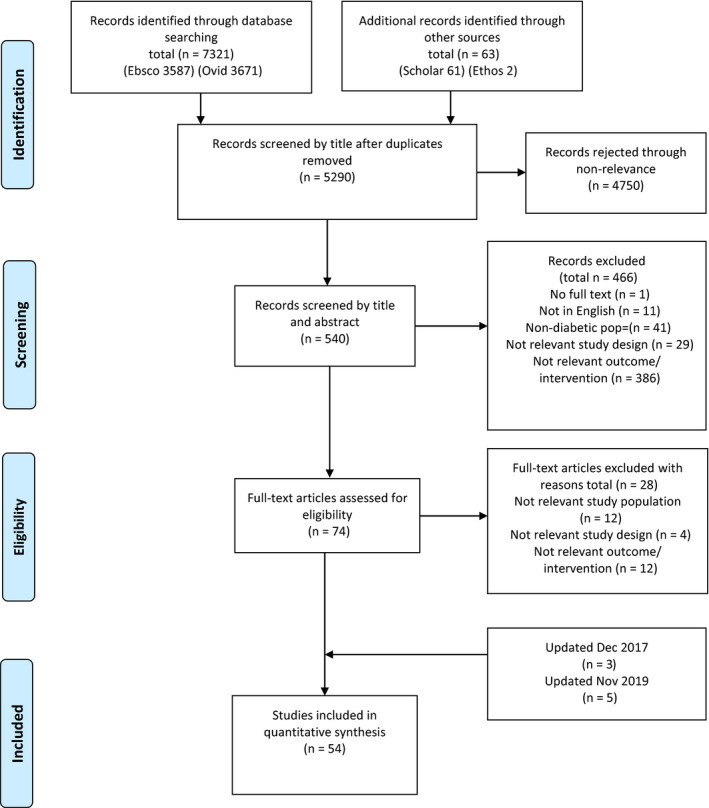
Flow diagram of study selection

The initial electronic search generated 7384 articles of which 2094 were duplicates (Figure 1). In the screening phase, 4750 were excluded based on their title and a further 466 excluded on title and abstract leaving 74 articles for full text assessment. We excluded 28 of these articles based on irrelevant study population (n = 12), irrelevant study design (n = 4), irrelevant outcome/ intervention (n = 12) leaving 46 ^20^, ^21^, ^22^, ^23^, ^24^, ^25^, ^26^, ^27^, ^28^, ^29^, ^30^, ^31^, ^32^, ^33^, ^34^, ^35^, ^36^, ^37^, ^38^, ^39^, ^40^, ^41^, ^42^, ^43^, ^44^, ^45^, ^46^, ^47^, ^48^, ^49^, ^50^, ^51^, ^52^, ^53^, ^54^, ^55^, ^56^, ^57^, ^58^, ^59^, ^60^, ^61^, ^62^, ^63^, ^64^, ^65^ included in the final review. As the initial search was undertaken in July 2016, updated searches were performed in December 2017 yielding 6918 articles, from which an additional three studies^66^, ^67^, ^68^ were included and November 2019 yielding 7821 articles from which a further five studies^69^, ^70^, ^71^, ^72^, ^73^ were included.

### 3.1 Data extraction

Data extraction of included studies was conducted using JBI Meta‐Analysis of Statistics: Assessment and Review Instrument (JBI‐MAStARI).^74^ In this phase, the general and contextual data were extracted in relation to the population, study design, interventions (features, design, modifications and materials of footwear and insoles) and outcomes. In addition, relevant information was extracted in the results section. Data extraction was carried out by (RC) and checked by the second reviewer (JP).

### 3.2 Data analysis and synthesis

In this review, we summarized study findings quantitatively and pooled study effects in a meta‐analysis when appropriate using JBI MAStARI.^74^ Meta‐analysis was performed using random‐effects models for continuous variables, calculating mean differences using the inverse variance method. Meta‐analysis was based on changes from baseline for peak pressure when the mean and SD were reported where any footwear or insole design feature, modification and material or method could be distinguished. Means and SD’s of data were required to be included in the meta‐analysis; we contacted four corresponding authors to request this data when not included in the article; two authors did not respond, and one no longer had access to the data.

For all estimates, we computed the 95% confidence intervals (CI’s). We quantified statistical heterogeneity using the *I*‐squared statistic (*I* ^2^) and considered heterogeneity as low (<25%), moderate (>25‐50%), or high (>50%),^75^ although we did not pre‐specify any degree of heterogeneity that would preclude meta‐analytic pooling.

### 3.3 Assessment of study quality

Two reviewers (RC and JP) independently assessed the methodological quality of the studies using the relevant JBI critical appraisal tools.^76^ Disagreements were resolved through consensus meeting. A study was considered low risk of bias if all criteria was included. Summaries of the appraisal of study quality are included in Appendix S2. All studies had some form of bias with standards of reporting variable across studies and by study design. From the quality assessment of the randomized controlled trials (RCT’s, all of the RCT studies had some form of bias (mean percentage of ‘yes’ scores = 65% ±SD 29%). All RCT studies reported inclusion criteria of participants, p values and participants lost to follow‐up. The most frequent omissions related to the blinding of the assessor and participants, concealing of treatment allocation and outcomes measurement. Within all of the cohort studies, some form of bias existed (mean percentage of ‘yes’ scores = 56% (±SD 31%). The most frequent omissions related to confounding factors, short follow‐up periods and incomplete follow‐up. Within the case‐controlled studies, mean percentage of ‘yes’ scores = 70% (±SD 0%). Omissions related to confounding factors, lack of sample size justification and different criteria used for the identification of cases and controls. For the case series study, percentage of ‘yes’ scores = 60%. Omissions related to inclusion criteria, reporting of demographics and participants’ characteristics. For the nonrandomized crossover study, percentage of ‘yes’ scores = 75% with omissions relating to confounding factors and selection bias.

### 3.4 Characteristics of included studies

Study characteristics are reported in Table 1. Fifty‐four studies met the inclusion criteria. Study designs included: n = 13 RCT’s,^23^, ^25^, ^31^, ^38^, ^42^, ^49^, ^55^, ^56^, ^61^, ^62^, ^70^, ^73^, ^77^ n = 37 cohort studies,^20^, ^21^, ^22^, ^24^, ^26^, ^27^, ^28^, ^29^, ^30^, ^32^, ^33^, ^34^, ^35^, ^36^, ^37^, ^39^, ^40^, ^41^, ^43^, ^45^, ^47^, ^48^, ^49^, ^51^, ^52^, ^53^, ^54^, ^57^, ^58^, ^59^, ^60^, ^64^, ^66^, ^67^, ^68^, ^71^, ^72^ n = 2 case‐control studies,^44^, ^63^ n = 1 nonintervention case series study ^46^ and n = 1 nonrandomized cross‐sectional over trial.^65^ Four authors reported results of the same study in different papers^21^, ^22^, ^39^, ^40^, ^45^, ^47^, ^49^, ^50^ and therefore results from these studies were described, but only one set of each results was used within any meta‐analysis. Studies were published between 1975 and 2019, undertaken in US (n = 17),^20^, ^24^, ^33^, ^35^, ^37^, ^42^, ^45^, ^46^, ^47^, ^48^, ^51^, ^54^, ^55^, ^58^, ^59^, ^62^, ^65^ UK (n = 10),^23^, ^30^, ^32^, ^49^, ^50^, ^67^, ^68^, ^71^, ^73^, ^77^ Netherlands (n = 7),^21^, ^22^, ^26^, ^27^, ^36^, ^52^, ^64^ Germany (n = 4),^28^, ^29^, ^44^, ^57^ Italy (n = 2),^56^, ^61^ Australia (n = 3),^25^, ^31^, ^53^ Taiwan (n = 3),^39^, ^40^, ^43^ Spain (n = 2),^34^, ^70^ Thailand (n = 2),^66^, ^72^ Austria (n = 1),^41^ Sweden (n = 1),^38^ Hong Kong (n = 1)^60^ and India (n = 1).^63^ The number of participants recruited to treatment groups ranged from seven to 298. Twenty‐seven studies (50%) recruited participants with diabetes mellitus and peripheral neuropathy whilst 19 studies (35%) recruited participants with diabetes mellitus, peripheral neuropathy and history of foot ulceration; a further two studies recruited participants with diabetes mellitus and peripheral arterial disease; three studies recruited participants with diabetes mellitus and classified at high risk of foot ulceration; two studies recruited participants with diabetes mellitus only; two studies recruited participants with diabetes mellitus, peripheral neuropathy and high forefoot pressures; one study recruited participants with diabetes mellitus, peripheral neuropathy and foot deformity; one study recruited participants with diabetes mellitus and foot callus; one study recruited participants with diabetes mellitus and taking insulin; one study recruited participants with diabetes mellitus and classified at low risk of foot ulceration. Follow‐up time periods ranged from no follow‐up to five years.

**Table 1:** Characteristics of included studies in the systematic review

Author/year	Study setting	Study design	Participants	Age/y (SD)	Gender Male:Female	Comparator	Follow‐up period	Outcomes
Abbott et al^77^	UK	RCT	N = 58 DPN with history of previous foot ulceration	Control group 67.1 (9.6); intervention group 59.1 (8.5)	51:7	No plantar pressure feedback provided	18 mo	68% ulcer free in control group and 78% in intervention group
Albert & Rinoie^20^	US	Cohort study	n = 8 DPN	67 (10.1)	Unknown	Without orthotic	3 mo	PPP↓ 30%‐40% under 1st MTPJ & medial heel 5%‐10% ↑Total contact area
Arts et al^21^	Netherlands	Cohort study	n = 85 DPN, recently healed plantar foot ulcer	62.6 (10.2)	70:15	Premodification	15 mo	PPP↓23% at target location; PPP↓ 13.5%‐24% by adding metatarsal bar or pad with replacement of top‐cover
Arts et al^22^	Netherlands	Cohort study	n = 171 DPN with recently healed ulcer	62.8 (10.2)	140:31	Barefoot	Unknown	PPP↓ 50%‐76% (deformed feet), 14%‐66% (nondeformed feet) 85% (previous ulcer location). 61% Successfully offloading below 200 kPa & 62% at previous ulcer site
Barnett^23^	UK	RCT	n = 102 DM	Orthoses group = 56 (20‐75) Cleron group 62 (18‐75)	68:35	3mm cleron flat insoles	6 mo	With orthoses: (22% MPPP↓, 16% Pressure‐time integral↓ & 11%↑mean Contact area); With insoles (16% ↓MPPP, 10% Pressure‐time integral↓ & 2%↑ mean Contact area)
Birke et al^24^	US	Cohort study	n = 19 DM with history of foot ulceration	60.2 (9.8)	11:8	Patients own CMI & footwear & no orthosis	n/a	Mean PPP↓55% (wearing own CMI & shoe vs without insoles). mean PPP↓ 36%‐39% (standard shoe wearing ¼ inch medium hardness poron vs shoe without orthoses)
Burns et al^25^	Australia	RCT	n = 61 DM with PAD & MSK pain.	Custom group = 67.6 (8.4) Sham group = 65.4 (10.3) (13.3)	37:24	Sham insole	8 wk	Whole foot Mean PPP↓(18% CMI vs 8% sham); Rearfoot Mean PP↓(27% CMI vs 4% sham); Midfoot Mean PPP↓ (7% CMI vs 4% sham); Forefoot mean PPP↓(16% CMI vs 10% sham)
Bus et al^27^	Netherlands	Cohort study	n = 20 DPN with foot deformity	64.4 (11.2)	13:7	0.95cm PPT flat insole	n/a	PPP↓16% & Force time integral↓ with CMI vs 8% with flat insole at 1st MTPJ
Bus et al^26^	Netherlands	Cohort study	n = 23 DPN	59.1 (12.6)	17:6	Pre‐ and post‐modification		All 35 ROI’s successfully optimised with average of 30% ↓ PPP
Busch & Chantelau^28^	Germany	Cohort study	n = 92 DPN with history of healed ulceration	64	49:43	Without footwear provided	19 mo (shoes) vs 5 mo (without shoes)	45% Absolute ulcer risk reduction for with shoes in 1st year
Chanteleau et al^29^	Germany	Cohort study	n = 50 DPN	59 (12)	31:19	With therapeutic footwear	25 mo	Foot lesions = 78% pre‐intervention vs 41% post‐intervention
Chapman et al^30^	UK/Germany	Cohort	n = 24 healthy & n = 24 people with DM	57 (8)	31:17	Control	n/a	Variations in apex angle: 14% maximum pressure↓(1st MTPJ) & pressure↑(heel) vs control. For variations in apex position: 39% maximum pressure↓ at 2‐4MTPJ vs control As rocker angle ↑ there was ↓ in PP (5th MTPJ) & ↑ in pressure (hallux)
Colagiuri et al^31^	Australia	RCT	n = 20 DM & with callus	Orthotic group 63 (10); podiatry group 69 (6)	5:15	Traditional treatment of callus	12 mo	Callus grade improved in 16/22 callus sites (orthotic treatment group); remained unchanged in 23/30 & 7 deteriorated (traditional treatment group)
Cumming & Bayliff^32^	UK	Cohort study	n = 20 DM with vascular or neurological impairment	68	unknown	No insole	1 wk	Mean total pressure: wearing insole (0.180 kg cm^−2^ s^−1^), no insoles (0.210 kg cm^−2^ s^−1^) Mean pressure redistribution Poron 96 (0.198 kg cm^−2^ s^−1^), Poron 4400 (0.211 kg cm^−2^ s^−1^); total difference (0.013 kg cm^−2^ s^−1^).
Donaghue et al^33^	US	Cohort study	n = 50 DM at high risk of foot ulceration	57.6 (34‐78)	32:18	Old footwear	3 & 6 mo	Peak force at baseline: socks only (6.15 kg cm^−2^), own socks & shoes (4.46 kg cm^−2^), new socks & shoes (3.98 kg cm^−2^). Mean PPP at 3 mo with new socks & shoes (4.13 kg cm^−2^) & 6 mo (4.24 kg cm^−2^)
Fernandez et al^34^	Spain	Cohort study	n = 117 DM with high risk foot factors & history of ulceration	Unknown	93:24	2 y pre‐intervention	Follow‐up 24 mo	Pre‐orthotic 147 ulcerations; post‐orthotic 22 ulcerations Mean PPP with orthotic treatment ↓ 85.2 kPa (left foot) & ↓87.6 kPa (right foot)
Frykberg et al^35^	US	Cohort study	n = 25 subjects (10DM, 15 healthy) with various foot shapes	37 (13.5)	13:12	Patients own tennis or oxford shoe	n/a	For DM subjects Mean PPP with: own shoe (4.46 kg cm^−2^), Surgical boot (4.89 kg cm^−2^), Surgical boot & rocker insole (2.50 kg cm^−2^). For nondiabetic subjects Mean PPP with: own shoe(2.07 kg cm^−2^), surgical boot (2.13 kg cm^−2^), Surgical boot & rocker insole (1.13 kg cm^−2^)
Guldemond et al^36^	Netherlands	Cohort study	n = 17 DPN nondeformed feet	Median 64 (44‐78)	Unknown	11 varying insoles	n/a	In central forefoot Mean PPP↓ with: metatarsal dome (32 kPa), standard arch (17 kPa), extra arch support (45 kPa). At medial forefoot Mean PPP↓ with: varus wedge (9 kPa), metatarsal dome (42 kPa), standard arch (12 kPa), extra arch support (38 kPa). At hallux Mean PPP↓ with extra arch & varus wedge (52 kPa)
Hastings et al^37^	US	Cohort study	n = 20 DPN	57.3 (9.3)	12:8	3 insole conditions	n/a	At 2nd MTPJ: PPP↓ (32%) when pad placed between 6.1 and 10.6 mm proximally; PPP ↓(16%) when pad located 1.8 mm distal to 6.1 mm proximally; PPP↓ (57%) when distal part of met pad was 10.6 mm proximal to met head; PPP↑ when pad was further than 1.8 mm distally or >16.8 mm proximally
Hsi et al^39^	Taiwan	Cohort study	n = 14 DPN	61.4 (8.3)	6:8	Patients’ own shoes	n/a	Diabetic footwear: pressure‐time integral (↓heel), (↓anterior to MTPJ), (↓at toe regions) (↑at the midfoot & posterior to MTPJ) PPP: (↓heel), (↓anterior to MTPJ), (↓at toe regions), (↑midfoot & posterior to MTPJ)
Hsi et al^40^	Taiwan	Cohort study	n = 10 DPN	63 (9)	3:7	Patients’ own shoes		Rocker sole ↓PPP & pressure‐time integral in anterior lateral, central lateral & central medial forefoot & prolonged time to PPP in posterior forefoot but not anterior forefoot
Kastenbauer et al^41^	Austria	Cohort study	n = 13 DM	56 (8)	5:8	Leather styled Oxford shoe	n/a	At great toe PPP ↓ with: cork insole & in‐depth shoe (16%), Adidas shoe(32%); CMI & in‐depth shoe (33%); At 1st MTPJ PPP ↓ with: cork insole & in‐depth shoe (27%), Adidas shoe(29%); CMI & in‐depth shoe (50%); At 2/3rd MTPJ PPP ↓ with: cork insole & in‐depth shoe (19%), Adidas shoe(47%); CMI & in‐depth shoe (48%); At heel PPP ↓ with: cork insole & in‐depth shoe (34%), Adidas shoe(34%); CMI & in‐depth shoe (39%).
Lavery et al^42^	US	Single physician blinded RCT	n = 299 DPN previous ulceration or neuropathy & foot deformity	Shear group 69.4 (10.0); Standard group 71.5 (7.9)	202:97	Insoles for standard treatment	18 mo	3.5 times odds of developing an ulcer; Three ulcers developed in shear resistant insole group, 10 ulcers developed in standard insole group
Lin et al^43^	Taiwan	Cohort study	n = 26 DPN	68 (9)	10:16	Standard shoe with insole	n/a	For regions of interest: 15.7% ↓Mean PPP (preplug removal); 32.3% ↓Mean PPP (pre‐ vs post‐plug removal); 14.3% ↓Mean PPP (arch addition to preplug removal vs post‐plug removal). For Non–regions of interest 8.7% ↓Mean PPP (preplug removal vs barefoot); 2.2% ↑Mean PPP with pre‐ vs post‐plug removal); 2.5% ↓Mean PPP (arch addition to preplug removal vs post‐plug removal).
Lobmann et al^44^	Germany	Case control	n = 81 type 2 DM (n = 18 DPN & high forefoot pressures vs n = 63 control)	Intervention group 63 (9); control group 66 (10)	Unknown	Neutral shoes	8 wk & 6 & 12 mo	32.6% ↓Maximum PPP at issue 28% ↓ Maximum PPP at 6 mo; 13% ↓ Maximum PPP at 12 mo
Lopez‐Moral et al^70^	Spain	RCT	N = 51DPN and previous foot ulceration	Intervention group 61 (8.1); control group 60 (8.6)	Intervention group 24:2; Control group 23:2	Semi‐rigid rocker	6 mo	Rigid rocker sole ↓ reulceration risk by 64%
Lott et al^45^	US	Cohort study	n = 20 DPN & history of ulceration	57.3 (9.3)	12:8	Barefoot	n/a	Mean applied pressure: barefoot (272 kPa); shoe (173 kPa), shoe & CMI (140 kPa); CMI & metatarsal pad, (98 kPa). Soft Tissue Strain at 2nd MTPJ: barefoot (38.2%), shoe (31.6%); shoe & CMI (28.9%); shoe, CMI & Metatarsal Pad (24.1%).
Martinez‐Santos et al^71^	UK	Cohort study	n = 60 DPN	67 (13)	40:20	Flat insole	n/a	PPP ↓ of 29 kPa with metatarsal bar and EVA/poron materials
Mohamed et al^46^	US	Case series comparison	n = 16 DPN Type 2 (n = 8 Plastazote vs n = 8 Plastazote/Aliplast)	Plastazote group 68.4 (5.5); Plastazote/Aliplast group 68.9 (5.5)	8:8	No insole	1 mo & 3 mo	With CMI at baseline: decrease in PPP (12.0 N cm^−2^); Max Mean Pressure (4.9 N cm^−2^); Pressure‐time integral (5.6 N cm^−2^ s^−1^) & ↑Total Contact Area (21.2 cm^2^) At follow‐up: decrease in PPP (10.5 N cm^−2^); Maximum mean pressure (5.2 N cm^−2^) & Pressure‐time Integral (5.9 N cm^−2^ s^−1^) & ↑ Total Contact Area (20.2 cm^2^).
Mueller et al^47^	US	Cohort study	n = 20 DPN & history of forefoot ulcer	57 (9)	12:8	Shoes with standard insoles	n/a	19%‐24% PPP↓ (CMI), 15%‐20% PPP↓ (CMI + metatarsal pad); 16%‐23% Pressure‐time Integral ↓ (with CMI), 22%‐32% Pressure‐time Integral↓ (CMI + metatarsal pad & shoe).
Nouman et al^66^	Thailand	Cohort study	n = 16 DPN	58 (9)	9:7	Without CMI	n/a	PPP↓26% at forefoot and 24% at toes with CMI
Nouman et al^72^	Thailand	Cohort Study	N = 16 DPN	Unknown	9:7	Addition of multifoam top cover	n/a	forefoot maximum PPP 248.2 kPa (61.92) with CMI; 211.6 kPa (47.01) with CMI and multifoam
Owings et al^48^	US	Cohort study	n = 22 DPN & high pressures (>750 kPa) in MTPJ region	63.7 (10.7)	11:11	Polypropylene shell with Korex sponge or plastazote cover; EVA shore 45 with procell or plastazote cover.	n/a	168 kPa PPP at regions of interest (shape‐based & pressure‐informed CMI); 211 kPa PP (shape‐based & 45 Shore EVA base with Procell or Plastazote top cover); 246 kPa PPP (polypropylene shell with Korex, sponge or plastazote top cover CMI); In rocker shoes: 127 kPa PPP at regions of interest (shape‐based & pressure‐informed CMI); 178 kPa PPP (shape‐based & 45 Shore EVA base with Procell or Plastazote top cover CMI); 200 kPa PP (shape‐based & polypropylene shell with Korex, sponge or plastazote top cover CMI).
Parker et al^73^	UK	RCT	n = 57DPN	Traditional group 61.4 (10), digital group 66.3 (10.5)	45:7	Control insole 3mm poron	6 mo	Compared with control insole PPP ↓14.91% with traditional insole and ↓24.43% with digital insole at baseline
Paton et al^50^	UK	RCT	n = 119 DPN	custom group 71 (10) prefab group 70 (10)	90:29	Prefabricated contoured shell	6 mo	With CMI (37% ↓PPP at baseline & 6 mo); (27% ↓Pressure‐time Integral at baseline & 30% at 6 mo); (32% ↑Total Contact Area baseline & 15% at 6 mo). With Prefabicated insole: (35% ↓PPP at baseline & 31% at 6 mo); (22% ↓Pressure‐time Integral & 24% at 6 mo); (29% ↑Total Contact Area at baseline & 15% at 6 mo); No difference between CMI and prefabricated insole in PPP & Total Contact Area
Paton et al^49^	UK	Observational cohort study	n = 60 DPN	69	47:22	Prefabricated contoured shell	3, 6,12 mo	↓PPP with CMI of 39% (0 mo), 35% (6 mo) & 36% (12 mo)
Perry et al^51^	US	Cohort study	n = 39 total: 13 DM, 13 DPN, 13 nondiabetic	DM group 53.6 (9.4); DPN group 52.8 (7.3); Nondiabetic group 54.2 (9.7)	33:6	Sock only	n/a	Oxford shoes vs socks: 18% ↓Mean PPP (2nd MTPJ), 2.3% ↓Mean PPP (MTPJ’s & heel); Running shoe vs socks 31% ↓Mean PPP (forefoot & heel)
Praet & Louwerens^52^	Netherlands	Cohort study	n = 10 DPN	63 (44‐78)	0:10	Oxford shoe without insole	n/a	3 Oxford type shoes show no significant ↓ in pressure vs baseline; rocker bottom shoes showed ~50% ↓PPP in central forefoot vs no rocker; mean ↑Total Contact Insole with insole (3.4‐7.3 cm^2^)
Preece et al^67^	UK	Cohort study	n = 102 DM at low risk and n = 66 healthy control	57 (9)	52:50	8 shoe conditions	n/a	Optimum location of 52% apex, 20° angle and apex 95°
Raspovic et al^53^	Australia	Cohort study	n = 8 DPN with past ulceration	61 (48‐68)	8:0	No insole	n/a	↓PPP, Pressure‐time Integrals & ↑Total Contact Area
Reiber et al^54^	US	Cohort study	n = 24 DPN no history of ulceration	66 (9.3)	Unknown	Preformed insole	Upto 6 mo	0 breaks in skin at 6 mo
Reiber et al^55^	US	RCT	n = 400 DM with history of foot ulceration	62	309:91	Usual footwear	2 y	Number of feet ulcerated 15% (shoes & cork insoles), 14% (shoes & prefabs), 17% (control group)
Rizzo et al^56^	Italy	RCT	n = 298 DM at high risk	Standard group 66.2 (9.4) intervention group 68.1 (14.1)	Unknown	Standard care	12 mo, 3 & 5 y	Foot ulceration development: At 12 mo 13% (intervention) vs 38.6% (standard care). At year 3, 18% (intervention) vs 61% (standard care); At year 5, 24% (intervention) vs 72% (standard care)
Sacco et al^57^	Germany	Cohort study	n = 45 participants (21 control, 24 DPN)	DPN group 55.2 (7.9) Control group 50.9 (7.3)	Unknown	barefoot	n/a	1st Ground Reaction Force peak > during shod conditions & > propulsion force in diabetic group but 2nd Ground Reaction Force peak < in shod diabetic vs control group
Scherer^58^	US	Cohort study	n = 7 insulin taking DM patients	38 (28‐59)	3:4	n/a	10 wk	Six patients discontinued use of footwear (five plantar irritation of heel & one hypertrophic lesions under 4/5th MTPJ’s)
Soulier ^59^	US	Cohort study	n = 108 DM Caucasian nonsmokers	55 (19‐55)	33:45	Own shoes	monthly	Significant change in callus size with running shoes
Tang et al^38^	Sweden	RCT	n = 114 DPN & previous ulceration	58 (15)	62:52	Prefabricated insole	2 y at 6 monthly	PPP = 180 kPa (35 EVA insole); 189 kPa (55 EVA insole); 211 kPa (prefab)
Telfer et al^68^	UK	Cohort study	n = 20 DPN	64.4 (9.2)	15:5	Barefoot	n/a	Optimized milled lowered PP by 41.3 kPa compared with CMI and optimised printed lowered PPP by 40.5 kPa compared with CMI
Tsung et al^60^	Hong Kong	Cohort study	n = 6 DPN vs n = 8 control	DPN group 56.2 (6.2); control group 46.5 (11.7)	Unknown	Shoe‐only	n/a	Mean PPP↓ 13.4% (Non‐weight‐bearing insole), 13.8% (Semi‐weight‐bearing insole), 8.1% (Fully weight‐bearing insole), 2.4% (flat insole)
Uccioli et al^61^	Italy	RCT	n = 69 high risk/past ulcer	Pod group 59.6 (11); Control 60.2 (8.2)	43:26	Nontherapeutic shoes	12 mo	Ulcer relapse 58.3% (control) vs 27.7% (intervention)
Ulbrecht et al^62^	US	RCT	n = 150 DPN recently healed ulcer	Experiment group 60.5 (10.1); Control group 58.5 (10.7)	104:46	Standard insoles	15 mo	Ulcer occurrence control > insole; no difference in nonulcerated lesion.
Viswanathan et al^63^	India	Case control	n = 241 DM previous foot ulceration	Gr1 = 59.1 (8.2); Gr2‐54.5 (9.1); Gr3 = 53.9 (9.3); Gr4 = 59.1 (11.7)	156:85	Usual footwear	9 mo	PPP↓ 57% (MCR insole); 61% (Polyurethane); 58% (moulded footwear) 39% (own shoe)
Waajiman et al^64^	Netherlands	Cohort study	n = 117 DPN (85 experimental vs 32 control)	63.3 (10.1)	Unknown	Pre‐ and post‐modification	3 monthly until 1 year	PPP↓ 23% (ulcer site) & 21% (highest PPP site)
Wrobel et al^65^	US	Cross‐sectional analysis	n = 27 DPN pre‐ulcer callus/past ulceration	65.1	14:13	Standard control insoles	n/a	↓Temperature of 64.1% (forefoot) & 48% (midfoot) with DFO

Abbreviations: CMI, custom‐made insole; DPN, diabetic peripheral neuropathy; DM, diabetes mellitus; MTPJ, metatarsal phalangeal joints; PPP, peak plantar pressure; US, United States; UK, United Kingdom; ↓, decrease; ↑, increase, n/a—not applicable.

### 3.5 Description of outcome measures

Twenty per cent (n = 11) of studies^29^, ^34^, ^42^, ^54^, ^55^, ^56^, ^58^, ^61^, ^62^, ^70^, ^77^ reported foot lesions and ulceration as the primary outcome measure. Measurement of this outcome varied across all of the studies, with only one study^54^ using a validated wound classification system; six studies^34^, ^42^, ^55^, ^62^, ^70^, ^77^ used a broad definition of ‘lack of skin integrity through loss of the epidermis and dermis’, and the remaining studies had no definition of an ulcer or lesion.^29^, ^56^, ^58^, ^61^ All of these studies used professional judgement to assess for the presence of ulceration, although two of the studies^55^, ^62^ used photographs as a means of blinded assessment. Four per cent (n = 2) studies^31^, ^59^ used the presence of callus as the primary outcome measure, one study^31^ applied a nonvalidated grading system to assess callus condition, whilst the other^59^ measured diameter and thickness of callus lesion. One study^57^ reported ground reaction force (GRF) and electromyographic (EMG) activity of three muscles as outcome measures. One study^65^ used temperature (°C) as an outcome measure, inferring a rise in temperature with increased risk status when testing the shear reduction device. Seventy‐two per cent (n = 39) of studies^20^, ^21^, ^22^, ^23^, ^24^, ^25^, ^26^, ^27^, ^30^, ^32^, ^33^, ^35^, ^36^, ^37^, ^38^, ^39^, ^40^, ^41^, ^43^, ^44^, ^45^, ^46^, ^47^, ^48^, ^49^, ^50^, ^51^, ^52^, ^53^, ^57^, ^60^, ^63^, ^64^, ^66^, ^67^, ^68^, ^71^, ^72^, ^73^ used kinetic outcomes to evaluate the effectiveness of the footwear and insole intervention provided. However, there was considerable inconsistency in the measures amongst these studies, with mean peak pressure, maximum pressure, maximum mean pressure, mean total pressure, pressure‐time integral and force‐time integral all used.

### 3.6 Profile/shape of the insole, shoe upper and shoe outsole

Two features of insole profile were described in the majority of studies; arch profile and rocker profile. In total, 69% (n = 37) of studies^20^, ^21^, ^22^, ^23^, ^24^, ^25^, ^26^, ^27^, ^28^, ^29^, ^34^, ^36^, ^37^, ^38^, ^41^, ^43^, ^44^, ^45^, ^46^, ^48^, ^49^, ^50^, ^51^, ^53^, ^54^, ^55^, ^56^, ^58^, ^59^, ^60^, ^61^, ^62^, ^63^, ^64^, ^66^, ^68^, ^73^ reported using an arch profile as a feature of an insole (Appendix S3) and 37% (n = 20) of studies^26^, ^28^, ^29^, ^30^, ^34^, ^35^, ^38^, ^40^, ^48^, ^49^, ^50^, ^52^, ^54^, ^55^, ^56^, ^61^, ^64^, ^65^, ^67^, ^70^ reported rockers as an added feature of the shoe outsole (Appendix S4). One study^39^ lacked enough clarity in the description of the intervention to determine whether a rocker feature was used in the diabetic footwear.

**Figure 2:**
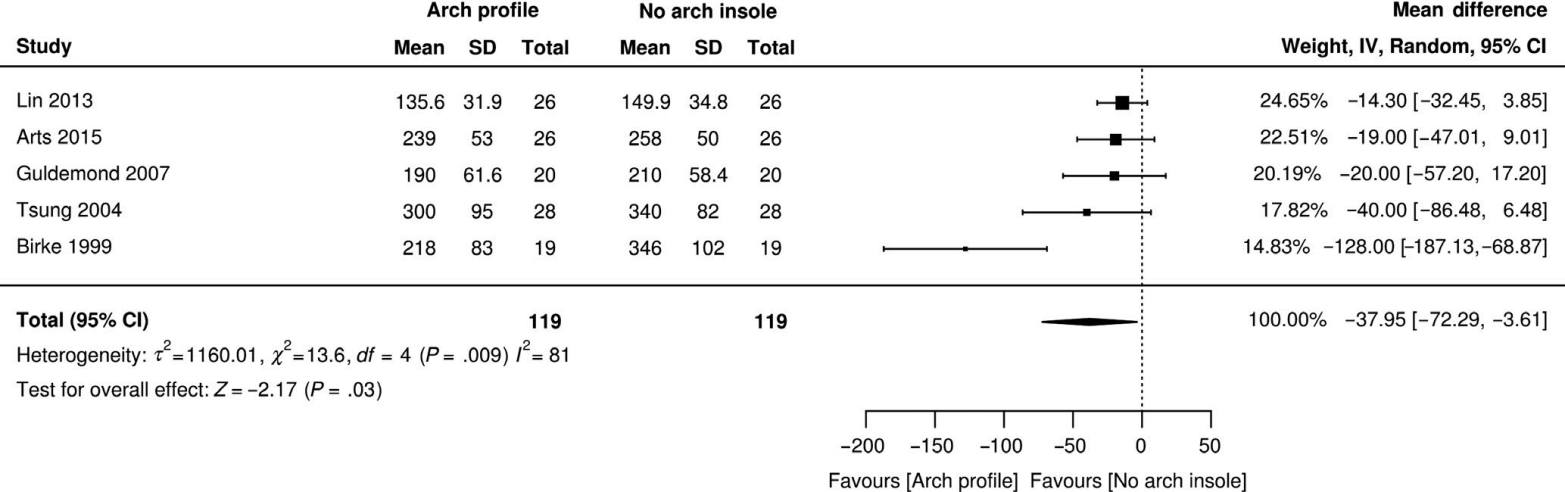
Forest plot of peak pressure for arch profile

Only 10% (n = 5) repeated measure studies ^21^, ^24^, ^36^, ^43^, ^60^ measured the direct effect of an arch profile on mean peak pressure. According to the heterogeneity test, high heterogeneity existed (*I* ^2^ = 81%, χ^2^ = 13.6, *τ* ^2^ = 1160, *P* = .009). Therefore, random‐effects modelling was applied to consolidate the effect value. Figure 2 shows that that out of 119 participants, the addition of an arch profile reduced peak pressure by a mean of 37 kPa (MD, −37.5; 95% CI, −72.29 to −3.61; *P* < .03) when compared to a flat insole. For the remaining 31 studies^20^, ^22^, ^23^, ^25^, ^26^, ^27^, ^28^, ^29^, ^34^, ^37^, ^38^, ^41^, ^44^, ^45^, ^46^, ^48^, ^49^, ^50^, ^51^, ^53^, ^54^, ^55^, ^56^, ^58^, ^59^, ^61^, ^62^, ^63^, ^64^, ^66^, ^68^ who reported using the arch profile as a feature of the insole, meta‐analysis was not conducted due to an inability to isolate the effect of this feature from other features of the insole.

Four studies reported the effect of a rocker profile. One study reported that in 71%‐81% of participants tested an optimum peak pressure target value of under 200 kPa could be achieved with a combination of apex position at 52% of shoe length and rocker angle of 20°.^67^ Another study reported no interaction effect when altering apex angle, apex position and rocker angle compared with the control shoe.^30^ A third study reported decreases in peak pressures and pressure‐time integrals in the posterior and anterior, central lateral and central medial forefoot with a standardized rocker shoe with apex position (83 mm on medial and 87 mm on lateral from front of shoe), angle thickness (24 mm maximum thickness at rocker with 11 mm rocker height at front end) compared to shoe without rocker.^40^ A fourth study reported ulcer reoccurrence to be 64% with a semi‐rigid rocker sole compared to 23% with a rigid rocker sole.^70^ There was an inability to distinguish the effect of the rocker profile feature from other features of the footwear and insole for those remaining studies.^26^, ^28^, ^29^, ^34^, ^35^, ^38^, ^48^, ^49^, ^50^, ^52^, ^54^, ^55^, ^56^, ^61^, ^64^, ^65^

### 3.7 Modifications made to the insole and shoe outsole

Sixty‐five per cent (n = 35) of studies^20^, ^21^, ^22^, ^24^, ^26^, ^31^, ^33^, ^34^, ^37^, ^39^, ^41^, ^43^, ^44^, ^49^, ^50^, ^52^, ^53^, ^54^, ^55^, ^56^, ^58^, ^60^, ^61^, ^62^, ^65^, ^70^ reported modification of footwear, although no separation of this feature from others would allow a pooled effect analysis to occur (Appendix S5). Fourteen studies^20^, ^21^, ^22^, ^24^, ^26^, ^34^, ^37^, ^41^, ^43^, ^52^, ^56^, ^60^, ^61^, ^62^ reported using extra‐depth shoes as a modification, five studies used diabetic footwear ^31^, ^39^, ^43^, ^49^, ^50^ and one study ^60^ reported patient‐specific footwear, customized to the individual, but did not report the effect this had on any outcome measure.

**Figure 3:**
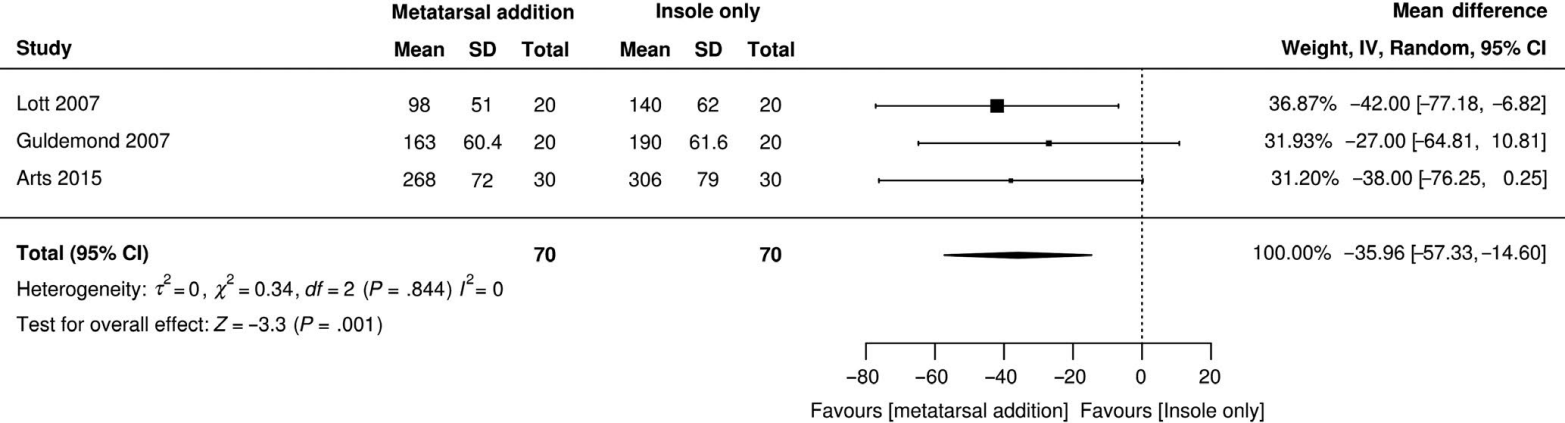
Forest plot of peak pressure for metatarsal modification

Thirty‐three per cent (n = 18) of studies^21^, ^22^, ^23^, ^26^, ^27^, ^36^, ^37^, ^38^, ^45^, ^46^, ^47^, ^48^, ^56^, ^62^, ^64^, ^68^, ^71^, ^73^ reported the use of metatarsal addition to the insole (Appendix S6). Only three repeated measure studies^21^, ^36^, ^45^ could distinguish the effect of a metatarsal addition independently from other insole and footwear features and were used for the meta‐analysis. According to the heterogeneity test, high heterogeneity existed (*I* ^2^ = 0%, χ^2^ = 0.34, *τ* ^2^ = 0, *P* = .844). Therefore, random‐effects modelling was applied to consolidate the effect value. Figure 3 shows that out of 70 participants, the use of a metatarsal addition in an insole reduced mean peak pressure by a further 35.96 kPa (MD, −35.96; 95% CI, −57.33 to −14.60; *P* < .001) when compared to an insole without metatarsal addition. There was a lack of description of the metatarsal addition, and no clear indication of how or when to utilize it as a modification.

Twenty‐two per cent (n = 12) of studies^21^, ^22^, ^26^, ^27^, ^34^, ^43^, ^48^, ^53^, ^64^, ^68^, ^70^, ^73^ modified insoles with the use of a cut out or aperture to target the site or lesion under the foot of clinical interest (Appendix S7). However, only two studies^21^, ^43^ reported the direct effect of this feature. Arts (2015) reported the reduction of in‐shoe peak pressure of 21 kPa from 253 (48) kPa to 232 (54) kPa with the removal of material in the insole for a variety of target locations^21^; and Lin reported reductions of MPP at regions of interest (ROI) located in the forefoot by 72 kPa from 221.4 (50.3) kPa to 149.9 (34.8) kPa with the removal of 1 cm × 1 cm^2^ plugs from underneath ROI.^43^

Thirteen per cent (n = 7) of studies^27^, ^31^, ^33^, ^36^, ^42^, ^73^, ^77^ used ‘other’ modifications. One study reported a 71% reduction on ulcer incidence when using ‘intelligent’ insoles with pressure detecting sensors compared to the control group.^77^ One study reported a 9 kPa reduction in mean peak pressure when adding a custom‐made five degree full length varus and valgus cork posts to the base of the insole for 20 participants with diabetic peripheral neuropathy and nondeformed feet.^36^ The remaining studies did not report the effect of these modifications. One study reported balancing the ¾ length orthotic with the use of dental acrylic posts at the rearfoot^31^ and another study used extra‐density padding at the heel, forefoot and covering the toes as a modification.^33^ Another study reported the use of wedge or medial skive on two occasions, prescribed at the discretion of an orthotist, but no rationale for use provided.^73^ One study reported including elastic binders and two nonstick sheets placed between the upper and lower pad of the insole as part of their shear resistant insole,^42^ and one study used substantial heel cups in the design of their insole, although no specification was disclosed.^27^

### 3.8 Fabrication techniques used for the insole and shoe

Forty‐three per cent (n = 23) of studies^20^, ^21^, ^22^, ^25^, ^26^, ^27^, ^31^, ^37^, ^38^, ^45^, ^48^, ^49^, ^50^, ^54^, ^55^, ^56^, ^60^, ^61^, ^63^, ^65^, ^66^, ^68^, ^72^, ^73^ used casting techniques to fabricate the insole and shoe (Appendix S8), and 20% (n = 11) of studies^21^, ^26^, ^27^, ^34^, ^36^, ^43^, ^48^, ^54^, ^56^, ^64^, ^73^ used kinetic information to inform the fabrication of the insole or shoe (Appendix S9). One study used both a ‘traditional’ foam box casting technique and a weight‐bearing foot scan technique.^73^ Another study^44^ used a pedorthist to prepare the insoles individually, although no further information was reported and one study^29^ reported the manufacture of the shoe by a local shoemaker according to an algorithm, but did not disclose the technique of the insole fabrication. Three studies^23^, ^49^, ^50^ used preformed insoles.

Only one repeated measures study^60^ reported effects of casting techniques to manufacture insoles under different loading conditions. Therefore, pooled analysis was not possible due to the diversity of techniques and lack of reported outcomes. Tsung et al^60^ reported decreases in MPP compared with shoe only condition of 13.4% when casted non‐weight‐bearing, 13.8% when casted with a semi‐weight‐bearing insole, 8.1% when casted with a full‐weight‐bearing insole, and 2.4% with a flat insole.

**Figure 4:**
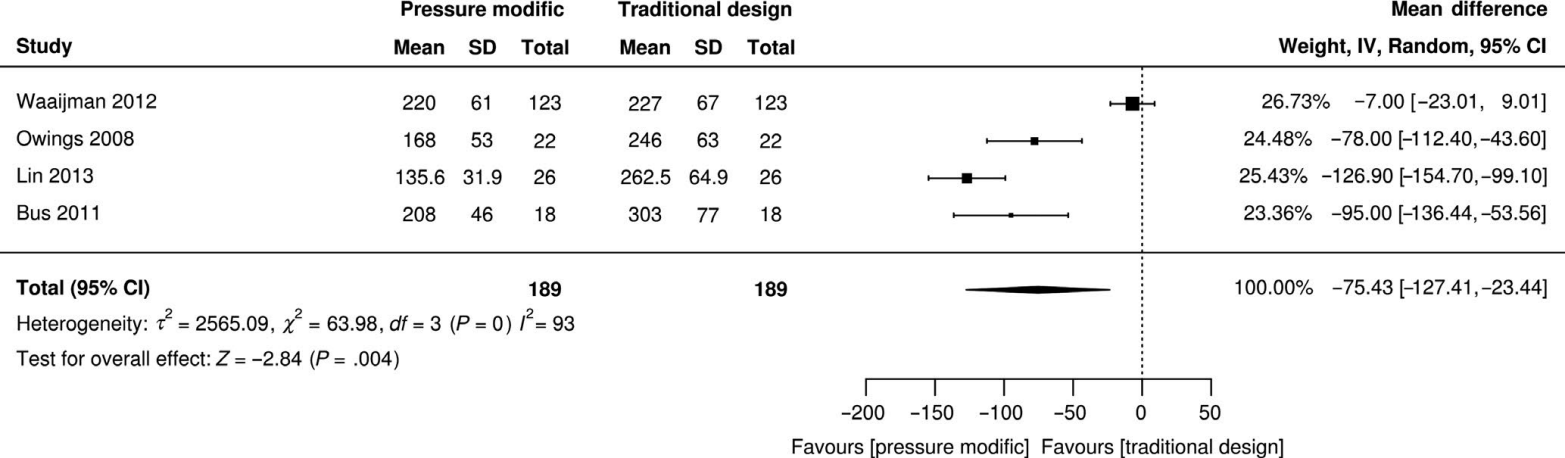
Forest plot of peak pressure for pressure designed

Twenty per cent (n = 11) of studies ^21^, ^26^, ^27^, ^34^, ^36^, ^43^, ^48^, ^54^, ^56^, ^64^, ^71^ used kinetic analysis to inform the design and modification of the insole (Appendix S9). Only one study^56^ used ulceration as an outcome measure, the remainder using kinetic measures. Four repeated measure studies^26^, ^43^, ^48^, ^64^ reported the direct effect of using plantar‐based pressure analysis as a fabrication technique to inform the design and modification of the insole and shoe in reducing mean peak pressure. According to the heterogeneity test, high heterogeneity existed (I^2^ = 93%, χ^2^ = 63.98, τ^2^ = 2565.09, *P* = 0). Therefore, random‐effects modelling was applied to consolidate the effect value. Figure 4 shows that in 189 participants, MPP in insoles fabricated with the use of an in‐shoe system was reduced by 75.4 kPa (MD, −75.4 kPa; 95% CI, −127.4 kPa to −23.44 kPa; *P* < .004) compared with those insoles fabricated using traditional techniques not involving pressure measurement systems.

### 3.9 Material type and properties of the insole and shoe outsole

Sixty‐nine per cent (n = 37) of studies^21^, ^22^, ^23^, ^25^, ^26^, ^27^, ^28^, ^29^, ^30^, ^34^, ^36^, ^41^, ^42^, ^43^, ^44^, ^46^, ^48^, ^49^, ^50^, ^52^, ^53^, ^54^, ^55^, ^56^, ^58^, ^60^, ^61^, ^62^, ^63^, ^64^, ^65^, ^66^, ^68^, ^70^, ^71^, ^72^, ^73^ used a combination of materials with diverse properties to manufacture the insoles or shoe outsole (Appendix S10). Thirty per cent (n = 16) of studies^20^, ^23^, ^27^, ^29^, ^34^, ^35^, ^46^, ^48^, ^49^, ^50^, ^52^, ^54^, ^55^, ^58^, ^60^, ^61^, ^62^, ^68^ used dual density constructs, thirty‐nine per cent (n = 21) of studies^21^, ^22^, ^25^, ^26^, ^28^, ^30^, ^36^, ^41^, ^42^, ^43^, ^44^, ^52^, ^53^, ^56^, ^63^, ^64^, ^65^, ^66^, ^70^, ^72^, ^73^ used tri or multi‐density/layers. Five studies examined the influence of material on reducing MPP. One RCT ^38^ of 114 DPN participants directly examined the effectiveness of CMI’s constructed of different materials. Comparisons of kinetic variables for a 35 shore ethyl‐vinyl acetate (EVA) CMI with a 55 shore hardness EVA CMI and a prefabricated insole (GloboTec, Comfort 312750501400) all within a standardized walking shoe were reported. The main pressure reduction between the CMI and the prefabricated insoles was achieved at the heel and in the overall peak pressure of 180 kPa with the extra soft durometer 35 shore hardness EVA insoles as opposed to 189 kPa for the soft 55 shore hardness EVA insole. The second study reported no statistical differences in reducing plantar pressures when comparing orthoses constructed of a single density material, Plastazote (Zotefoams Inc) with a dual density material, Plastazote and Alliplast (Voltek, Brennia, VA).^46^ The third repeated measures study reported a significant difference in MPP between different densities of poron in walking conditions (*P* < .0001) ^24^ although another study found no difference between Poron 96 and Poron 4000 in reducing peak pressure.^32^ A fifth study reported the reduction of maximum peak pressure at the forefoot with the addition of a multifoam top cover onto the dual density custom‐made insole of plastazote and microcellular rubber.^72^

## 4 DISCUSSION

The aim of this review was to identify the best footwear and insoles design feature for offloading the plantar surface of the foot to prevent foot ulceration in people with diabetes. More specifically, the objectives were to identify the key design features of footwear and insoles with regard to profile and shape, material type and properties, modifications and fabrication techniques.

Heterogeneity was found amongst the profile, modifications, material and fabrication techniques used in insoles and footwear design. Footwear and insoles can be viewed as multifaceted interventions where several features are frequently incorporated into the design. The studies highlighted the lack of a systematic approach to combining these features which makes it difficult to distinguish the effectiveness of individual features in offloading plantar foot pressures.

Within the review, we revealed variations in outcome measures, study design and quality. Six different outcome measures were used amongst the studies which makes meaningful comparison difficult. Identification of specific design features of footwear and insoles related to the primary outcome measure of foot ulceration was not possible. This was because all of the studies using foot ulceration as the outcome measure employed a combination of footwear and insole design features. The follow‐up time points at which outcomes were measured varied considerably across studies. The methodological quality of the studies was generally poor. Only four studies ^21^, ^38^, ^50^, ^73^ reported adherence to the insoles and footwear with one study excluding participants from analysis where there was a lack of substantial wear.^73^ The inclusion criteria contained participants with diabetes who were at different stages of disease progression, further adding to the difficulty in making meaningful comparisons between studies. Some studies included people with no sensory neuropathy; some studies included those with sensory neuropathy and no previous foot ulceration and some studies included participants with sensory neuropathy and previous foot ulceration. Foot complication severity has been shown to be associated with increased plantar foot pressures.^10^ However, this did not appear to influence the footwear or insole feature used.

### 4.1 Profile/shape of the insole, shoe upper and shoe outsole

Two types of profile features were described in this review; an arch and a rocker. The use of an arch profile replicating the contour of the plantar surface of the foot has traditionally been the ‘gold‐standard’ for insole design for reducing pressure in the diabetic neuropathic foot.^27^ This review found that 98% of studies reported using an arch profile as part of the insole configuration, although inconsistency exists in the reporting of the specifications. Our meta‐analysis provides evidence that an arch profile when added to an insole can enhance the offloading effect by a further 37 kPa when compared to an insole without an arch profile. It is postulated that by increasing contact with the plantar surface of the foot, thereby allowing an increased distribution of force over a greater area of the foot, plantar foot pressure will be reduced.^78^ Our review demonstrated that seven studies incorporating an insole with an arch profile reported that an increase in surface contact area values correlates with reduced forefoot pressures.^20^, ^23^, ^46^, ^49^, ^50^, ^53^, ^60^ However, Paton et al reported that the increase in total contact area observed at issue, reduced by 50% after 6 months of insole wear, whilst pressure reduction remained constant.^49^, ^50^ The authors suggest that this could be attributed to the dynamic nature of gait and associated pressure reduction may be associated with changes in foot function, such as the prevention of foot pronation.^79^, ^80^

Nineteen studies modified the rocker profile of the shoe as a method of reducing peak pressure. The rigid sole added to the bottom of the shoe is designed to limit the movement at foot joints, particularly extension of the metatarsophalangeal joints at the propulsive phase of gait. This prevents movement of tissue across the plantar aspect of the foot and alters the forefoot loading pattern, specifically reducing pressure under the metatarsal heads by 30%‐50%.^81^, ^82^ Our review demonstrates the multiplicity of design variables in terms of rocker angle, placement, height and material. Preece et al,^67^ suggested an optimum design of a rocker, but reported further adjustments of rocker angle and position reduced pressure on the forefoot across the participants. Chapman et al^30^ reported high inter‐subject variability for apex position in reducing pressure under the 1st MTPJ and hallux regions with no clear optimal position. Some consistency was achieved with reducing pressure under the 2nd to 4th MTPJ with an apex position of 50%‐60% of shoe length. The use of a rocker profile could be beneficial in reducing peak pressure under the diabetic neuropathic foot. However, the effectiveness of this feature may correlate with an individualized approach in the design of the rocker angle, placement, height and material, although no such design algorithm has yet been established.

### 4.2 Modifications

The purpose of modifications is to further adapt the footwear and insole by additional features. Three key modifications of insole and footwear design features were identified from this review; extra‐depth footwear, metatarsal additions and sinks or apertures. However, the inability to distinguish the effect of individual modifications from other insole and design features for the majority of studies creates uncertainty on the effectiveness of their usage. Additionally, the assortment of each modification with variations in design, materials, placement and fabrication made direct comparison extremely difficult. Despite this heterogeneity meta‐analyses verified the positive effect of metatarsal pad, cut‐outs or apertures in reducing forefoot plantar pressures. However, the effectiveness in reducing plantar pressure varies considerably with placement of the modification. For example, Hastings et al,^37^ established a pattern of increases or decreases in MPP according to placement of the metatarsal pad proximal or distal to the metatarsal, although only an effect on the 2nd metatarsal head was observed. A data‐driven approach using real‐time plantar pressure feedback, as utilized by 10 studies,^21^, ^26^, ^27^, ^34^, ^36^, ^43^, ^48^, ^54^, ^56^, ^64^ intimates that the effectiveness of some modifications could be enhanced by more accurate siting using appropriate technology, such as real‐time pressure analysis.

### 4.3 Fabrication techniques used for the insole and shoe

Two different fabrication techniques for insoles and footwear were identified in this review; casting and kinetic informed. Casting is traditionally used to capture the geometric shape of the patient's’ foot to ‘customize’ the insole. Only one study examined the role of three types of casting technique in reducing peak pressure.^60^ The authors reported an insole formed from a semi‐weight‐bearing foot shape offered the greatest peak pressure reduction compared with full‐weight‐bearing and non‐weight‐bearing foot shapes, but was not statistically significant. The remaining studies using a casting approach were not able to report any difference in reducing pressure using this fabrication method. This method of fabrication is believed to create an arch profile, which has been demonstrated as altering pressures in the plantar foot as reported by four studies.^21^, ^24^, ^36^, ^60^ However, one author, Paton et al,^50^, demonstrated no difference in reducing MPP and PTI when using a prefabricated insole compared with a customized insole. Therefore, potentially all insoles with an arch profile, regardless of the casting technique employed, are effective in reducing plantar pressure in people with diabetes. This view complements another finding of this review that suggests an arch profile may optimize the effect of insoles for diabetic feet.

Ten studies^21^, ^26^, ^27^, ^34^, ^36^, ^43^, ^48^, ^54^, ^56^, ^64^ reported the effect of using in‐shoe pressure measurement analysis to guide the fabrication of the footwear and insole. The use of a data‐driven approach for insole and footwear design has been heralded as authenticating plantar foot pressure reduction on an individual basis. Identification of the vulnerable plantar areas with pressure mapping, guides the design and alteration of appropriate personalized footwear and insoles in terms of materials, geometry and modifications. In addition, it provides a quantitative assessment of clinical outcome such that clinicians can be certain of achieving the desired treatment objective. Our meta‐analysis supports this proposition although variations in methodology with this technique requires a more consistent approach to limit the inconsistency across clinical areas. Only one study^54^ used pressure data to inform the design of the insoles; the remainder used the kinetic data to inform the modification of the insoles by iteratively testing and retesting until optimization was reached. A lack of standardization existed across all of the studies for temporal‐spatial measurements and gait parameters contributing to the analysis. The use of different pressure analysis systems with dissimilar technical specifications and resolution provides additional inconsistency. Furthermore, it should be acknowledged that foot plantar pressure values are only considered a surrogate measure of foot ulceration risk and that no threshold for foot ulceration has yet been established.^83^

### 4.4 Material type and properties of the insole and shoe outsole

Material choice is an important feature of any insole or footwear design. The material used, dependent on its mechanical and physical properties, will influence the insole or footwear's ability to redistribute or dampen forces effectively. This review found no consistency with individual materials used or thickness in the construction of footwear or insole. Only one study directly assessed the effect of material hardness in reducing peak plantar pressures.^38^ Sixty‐seven per cent of remaining studies used either dual or multi‐density material constructions of footwear and insoles. Closed cell foam materials were most frequently sited at the interface between foot and insole and footwear as a top cover; denser materials constituted the base of the insole, EVA appearing the most popular material of choice for the base. A less popular material type was thermoplastics, potentially because these materials were traditionally used for functional devices aimed towards changing gait function and not reducing pressure. Combining materials of different properties is suggested as incorporating the desired properties from each material to best serve reduction in foot ulceration risk.^84^, ^85^, ^86^ However, the literature does not provide a sufficiently robust evidence base to inform the selection approach regarding material combination or thickness for the best offloading. Therefore, selection of materials is often influenced by the availability of materials locally and anecdotal evidence, rather than patient‐specific characteristics and effectiveness of offloading.

## 5 LIMITATIONS

The primary limitation of this review is the heterogeneity of study design and outcome measures of the studies included. Large variations in the description of footwear and insoles and uncertainty in the reliability and validity of the assessment and intervention methods exists. The diversity of features used limits the generalizability of the results, resulting in variation in the number of studies and participants included within the meta‐analyses. This review was further limited by the inclusion of only English language studies, not including trial databases in the search database and exclusion of participants with charcot and foot amputation.

## 6 RECOMMENDATIONS

A consensus is required regarding how to report and measure the effectiveness of individual insole and footwear features in offloading the DPN foot. A core set of outcome measures and standardized time points would facilitate pooling of results in meta‐analyses to enable more accurate conclusions to be drawn. Standardization of inclusion criteria is further required to ensure all participants enrolled in offloading trials of DPN have DPN. This would also include participants with charcot and foot ulceration. Improved consistency in the reporting of methodology, in line with the Consolidated Standards of Reporting Trials guidelines and International working group on the diabetic foot, is also recommended.^83^

## 7 CONCLUSION

This systematic review highlights the difficulty in differentiating insole and footwear features in offloading the neuropathic diabetic foot. The amalgamation of features in insole and footwear designs makes consolidation of the body of knowledge difficult for understanding which feature to use at which time point. However, on the basis of this review, we conclude that metatarsal additions, apertures and arch profiles are effective in reducing plantar pressure in this population and therefore should be incorporated as footwear and insole features. Different casting techniques and materials also appear effective in reducing pressures, but we are unable to recommend any particular technique or type because of insufficient evidence. The use of pressure analysis to enhance the effectiveness of the design of footwear and insoles, particularly through modification, is recommended, specifically in patients with diabetes and peripheral neuropathy.

## CONFLICTS OF INTEREST

Richard Collings is funded by a National Institute for Health Research (NIHR) Clinical Doctoral Fellowship for this research project. This publication presents independent research funded by the National Institute for Health Research (NIHR). The views expressed are those of the author(s) and not necessarily those of the NHS, the NIHR or the Department of Health and Social Care.

## AUTHORS' CONTRIBUTION

RC, JF, JML and JP conceived and designed the study. RC designed the search string. RC and JP performed the literature search, assessed the literature, extracted data and drew conclusions. RC wrote the manuscript. JF, JML and JP critically reviewed and edited the manuscript. All authors have read and approved the final manuscript.

## ETHICS APPROVAL

This review manuscript summarizes and informs of already published studies and thus does not require ethical approval.

## Supporting information

Appendix S1

Appendix S2

Appendix S3

Appendix S4

Appendix S5

Appendix S6

Appendix S7

Appendix S8

Appendix S9

Appendix S10

## Data Availability

The data that support the findings of this study are available from the corresponding author upon reasonable request.
