# Supplementary material for: Footwear and insole design features for offloading the diabetic at risk foot—A systematic review and meta‐analyses
Source: Endocrinol Diabetes Metab. 2020 Apr 11;4(1):e00132. doi: 10.1002/edm2.132 (PMC7831212; doi:10.1002/edm2.132)
Supplement: Supplementary file 1 — Appendix S1 [file EDM2-4-e00132-s001.docx]

Electronic supplement material 1 – example of search string

"(((diabet*).ti,ab OR (diabetes mellitus).ti,ab) AND ((foot).ti,ab OR (feet).ti,ab OR (neuropath*).ti,ab OR (ulcer*).ti,ab OR (pressure).ti,ab OR (gait).ti,ab OR (walking).ti,ab)) AND ((time).ti,ab OR (offload*).ti,ab OR (off-load*).ti,ab OR (insole*).ti,ab OR (orthos*).ti,ab OR (orthotic devices).ti,ab OR (therapeutic footwear).ti,ab OR (shoes).ti,ab OR (shoe inserts).ti,ab OR (footwear).ti,ab OR (footwear intervention*).ti,ab OR (footwear adaption*).ti,ab OR (padding).ti,ab OR (plug*).ti,ab OR (ankle foot orthos*).ti,ab OR (offloading device*).ti,ab OR (rocker bottom).ti,ab OR (rocker sole*).ti,ab OR (flange*).ti,ab OR (arch profile).ti,ab OR (post*).ti,ab OR (skive).ti,ab OR (metatarsal bar).ti,ab OR (kinetic wedge).ti,ab OR (cut out).ti,ab)"
