# Supplementary material for: Footwear and insole design features for offloading the diabetic at risk foot—A systematic review and meta‐analyses
Source: Endocrinol Diabetes Metab. 2020 Apr 11;4(1):e00132. doi: 10.1002/edm2.132 (PMC7831212; doi:10.1002/edm2.132)
Supplement: Supplementary file 2 — Appendix S2 [file EDM2-4-e00132-s002.docx]

| Electronic supplementary material 2 -Quality appraisal of included studies | | | | | | | | | | | | | |
| --- | --- | --- | --- | --- | --- | --- | --- | --- | --- | --- | --- | --- | --- |
| Quality appraisal of randomized controlled trials | | | | | | | | | | | | | |
|  | Q1 Was true randomization used for assignment of participants to treatment groups? | Q2 Was allocation to treatment groups concealed? | Q3 Were treatment groups similar at the baseline? | Q4 Were participants blind to treatment assignment? | Q5 Were those delivering treatment blind to treatment assignment? | Q6 Were outcomes assessors blind to treatment assignment? | Q7 Were treatments groups treated identically other than the intervention of interest? | Q8 Was follow up complete and if not, were differences between groups in terms of their follow up adequately described and analyzed? | Q9 Were participants analysed in the groups to which they were randomized? | Q10 Were outcomes measured in the same way for treatment groups? | Q11 Were outcomes measured in a reliable way? | Q12 Was appropriate statistical analysis used? | Q13 Was the trial design appropriate, and any deviations from the standard RCT design (individual randomization, parallel groups) accounted for in the conduct and analysis of the trial? |
| Abbott et al, 2019 ^77^ | Y | N | N | N | N | N | Y | Y | Y | Y | Y | Y | Y |
| Barnett 2002 ^23^ | Y | N | Y | Y | Y | Y | Y | Y | Y | Y | Y | Y | Y |
| Burns et al, 2009 ^25^ | Y | Y | Y | Y | N | U | Y | Y | Y | Y | Y | Y | Y |
| Colagiuri et al, 1995 ^31^ | Y | N | Y | N | N | Y | Y | N | Y | Y | U | Y | U |
| Hellstrand Tang et al, 2014 ^38^ | Y | U | Y | Y | U | U | Y | Y | Y | Y | Y | Y | Y |
| Lavery et al, 2012 ^42^ | U | Y | Y | N | N | U | Y | Y | Y | Y | U | Y | Y |
| Lopez-Morales et al, 2019 ^70^ | Y | N | N | N | N | N | Y | Y | Y | Y | U | Y | Y |
| Parker et al, 2019 ^73^ | Y | N | Y | N | N | N | Y | N | N | Y | Y | Y | Y |
| Paton et al, 2012 ^50^ | Y | N | Y | Y | N | Y | Y | Y | Y | Y | Y | Y | Y |
| Reiber et al, 2002 ^55^ | Y | Y | Y | N | U | Y | N | U | Y | Y | Y | Y | Y |
| Rizzo et al, 2012 ^56^ | Y | N | Y | N | N | U | Y | N | Y | Y | N | N | Y |
| Uccioli et al, 1995 ^61^ | U | U | Y | U | U | U | Y | Y | Y | Y | U | N | Y |
| Ulbrecht et al, 2014 ^62^ | Y | Y | Y | U | U | Y | Y | Y | Y | Y | Y | Y | Y |
| % | 85 | 31 | 85 | 31 | 8 | 38 | 92 | 69 | 92 | 100 | 62 | 85 | 92 |

| Quality appraisal of cohort studies | | | | | | | | | | | |
| --- | --- | --- | --- | --- | --- | --- | --- | --- | --- | --- | --- |
|  | Q1 Were the two groups similar and recruited from the same population? | Q2 Were the exposures measured similarly to assign people to both exposed and unexposed groups? | Q3 Was the exposure measured in a valid and reliable way? | Q4 Were confounding factors identified? | Q5 Were strategies to deal with confounding factors stated? | Q6 Were the groups/participants free of the outcome at the start of the study (or at the moment of exposure)? | Q7 Were the outcomes measured in a valid and reliable way?7 | Q8 Was the follow up time reported and sufficient to be long enough for outcomes to occur? | Q9 Was follow-up complete, and if not, were the reasons to loss to follow-up described and explored? | Q10 Were strategies to address incomplete follow-up utilized? | Q11 Was appropriate statistical analysis used? |
| Albert & Rinoie 1994 ^20^ | Y | Y | N | N | N | Y | Y | N | Y | N/A | Y |
| Arts et al, 2012 ^22^ | Y | Y | Y | Y | Y | Y | Y | N | Y | N/A | Y |
| Arts et al, 2015 ^21^ | Y | Y | Y | Y | U | Y | Y | Y | Y | N/A | N |
| Birke et al, 1999 ^24^ | Y | Y | Y | Y | Y | Y | Y | N | Y | N/A | Y |
| Bus et al, 2004 ^27^ | Y | Y | Y | Y | Y | U | Y | N/A | Y | N/A | Y |
| Bus et al, 2011 ^26^ | Y | Y | Y | N | N | Y | Y | N | Y | N/A | N |
| Busch & Chantelau, 2003 ^28^ | Y | Y | Y | U | U | Y | U | Y | Y | U | Y |
| Chantelau 1990 ^29^ | Y | Y | U | N | N | Y | Y | Y | N | U | N |
| Chapman et al, 2013 ^30^ | N | Y | Y | N | N | Y | Y | N | Y | N/A | N |
| Cumming et al, 2011 ^32^ | Y | Y | Y | N | N | Y | Y | N | Y | N/A | Y |
| Donaghue et al, 1996 ^33^ | U | Y | U | U | U | U | Y | N/A | Y | N/A | Y |
| Fernandez et al, 2013 ^34^ | N | Y | N | Y | Y | Y | N | Y | U | N | N |
| Frykberg et al, 2002 ^35^ | N | Y | Y | N | N | U | Y | N/A | Y | N/A | N |
| Guldemond et al, 2007 ^36^ | N | Y | Y | N | N | Y | Y | N | Y | N/A | N |
| Hastings et al, 2007 ^37^ | U | U | Y | N | N | Y | Y | N | Y | N/A | Y |
| Hsi et al, 2004 ^40^ | Y | Y | Y | Y | Y | Y | Y | N | Y | N/A | Y |
| Hsi et al, 2002 ^39^ | Y | Y | Y | Y | Y | Y | Y | N | Y | N/A | Y |
| Kastenbauer et al, 1998 ^41^ | Y | Y | N | N | N | Y | Y | N | Y | N/A | Y |
| Lin et al, 2013 ^43^ | Y | Y | Y | U | U | Y | Y | N | Y | N/A | Y |
| Lott et al, 2007 ^45^ | Y | N | Y | Y | N | Y | Y | N | Y | N/A | N |
| Martinez-Santos et al, 2019 ^71^ | Y | Y | Y | N | N/A | Y | Y | N/A | N/A | N/A | Y |
| Mueller et al, 2006 ^47^ | Y | Y | U | N | N | Y | Y | N | Y | N/A | Y |
| Nouman et al, 2017 ^66^ | Y | Y | Y | U | U | U | Y | N | Y | N/A | Y |
| Nouman et al, 2019 ^72^ | Y | Y | Y | N | N | Y | Y | N | N/A | N/A | Y |
| Owings et al, 2008 ^48^ | N | Y | Y | U | N | Y | Y | N | Y | N/A | N |
| Paton et al, 2014 ^49^ | Y | Y | Y | Y | Y | Y | Y | Y | Y | N | N |
| Praet & Louwerens 2003 ^52^ | N | Y | Y | U | U | Y | Y | N | Y | N/A | N |
| Perry et al, 1995 ^51^ | N | Y | Y | Y | Y | U | Y | N | Y | N/A | Y |
| Preece et al, 2017 ^67^ | Y | Y | Y | U | U | Y | Y | N | Y | N/A | Y |
| Raspovic et al, 2000 ^53^ | N | Y | Y | N | N | Y | Y | N | Y | N/A | N |
| Reiber et al, 1997 ^54^ | N | Y | Y | N | N | U | U | Y | N | N | N |
| Sacco et al, 2010 ^57^ | N | Y | Y | N | N | Y | Y | N | Y | N/A | Y |
| Scherer 1975 ^58^ | Y | N | N | N | N | U | N | Y | Y | N/A | N |
| Soulier. 1986 ^59^ | U | Y | N | N | N | Y | Y | Y | Y | N | Y |
| Telfer et al, 2017 ^68^ | Y | Y | Y | N | N | Y | Y | N | Y | N/A | Y |
| Tsung et al, 2004 ^60^ | N | Y | U | N | N | Y | Y | N | Y | N/A | N |
| Waaijman et al, 2012 ^64^ | Y | Y | Y | Y | Y | Y | Y | N | Y | N/A | Y |
| % | 62 | 92 | 76 | 30 | 24 | 81 | 89 | 22 | 86 | 0 | 59 |

| Quality appraisal of case controlled studies | | | | | | | | | | |
| --- | --- | --- | --- | --- | --- | --- | --- | --- | --- | --- |
|  | Q1 Were the groups comparable other than the presence of disease in cases or the absence of disease in controls? | Q2 Were cases and controls matched appropriately? | Q3 Were the same criteria used for identification of cases and controls? | Q4 Was exposure measured in a standard, valid and reliable way? | Q5 Was exposure measured in the same way for cases and controls? | Q6 Were confounding factors identified? | Q7 Were strategies to deal with confounding factors stated? | Q8 Were outcomes assessed in a standard, valid and reliable way for cases and controls? | Q9 Was the exposure period of interest long enough to be meaningful? | Q10 Was appropriate statistical analysis used? |
| Lobmann et al, 2001 ^44^ | Y | Y | N | Y | Y | U | N | Y | Y | Y |
| Viswanathan et al, 2004 ^63^ | Y | Y | Y | Y | Y | N | N | Y | Y | N |
| % | 100 | 100 | 50 | 100 | 100 | 0 | 0.0 | 100 | 100 | 50 |

| Quality appraisal of case series study | | | | | | | | | | |
| --- | --- | --- | --- | --- | --- | --- | --- | --- | --- | --- |
|  | Q1 Were there clear criteria for inclusion in the case series? | Q2 Was the condition measured in a standard, reliable way for all participants included in the case series? | Q3 Were valid methods used for identification of the condition for all participants included in the case series? | Q4 Did the case series have consecutive inclusion of participants? | Q5 Did the case series have complete inclusion of participants?5 | Q6 Was there clear reporting of the demographics of the participants in the study? | Q7 Was there clear reporting of clinical information of the participants? | Q8 Were the outcomes or follow up results of cases clearly reported? | Q9 Was there clear reporting of the presenting site(s)/clinic(s) demographic information? | Q10 Was statistical analysis appropriate? |
| Mohamed et al, 2004 ^46^ | Y | Y | Y | U | U | N | N | Y | Y | Y |
| % | 100 | 100 | 100 | 0 | 0 | 0 | 0 | 100 | 100 | 100 |

| Quality appraisal for analytical cross-sectional study | | | | | | | | |
| --- | --- | --- | --- | --- | --- | --- | --- | --- |
| Citation | Q1 Were the criteria for inclusion in the sample clearly defined? | Q2 Were the study subjects and the setting described in detail? | Q3 Was the exposure measured in a valid and reliable way? | Q4 Were objective, standard criteria used for measurement of the condition? | Q5 Were confounding factors identified? | Q6 Were strategies to deal with confounding factors stated? | Q7 Were the outcomes measured in a valid and reliable way? | Q8 Was appropriate statistical analysis used? |
| Wrobel et al, 2014 ^65^ | Y | Y | Y | Y | U | U | Y | Y |
| % | 100 | 100 | 100 | 100 | 0 | 0 | 100 | 100 |
