# Supplementary material for: Footwear and insole design features for offloading the diabetic at risk foot—A systematic review and meta‐analyses
Source: Endocrinol Diabetes Metab. 2020 Apr 11;4(1):e00132. doi: 10.1002/edm2.132 (PMC7831212; doi:10.1002/edm2.132)
Supplement: Supplementary file 3 — Appendix S3 [file EDM2-4-e00132-s003.docx]

| Electronic supplementary material 3 – profile of insole | | |  |
| --- | --- | --- | --- |
| Profile of insole | Studies (n=37) | Comparator | Comments |
| Flat insole (non-moulded insoles) and medial longitudinal arch profile(CMI) | Birke et al, 1999^24^ | Non moulded insoles of different density materials in extra depth shoe | No specifications reported for non-moulded or CMI |
| Three types of CMI with medial longitudinal arch profile | Tsung et al, 2004  ^60^ | Different casting techniques to create digital image for CMI to individualise profile | Standardised adjustment technique in manufacture process, no specification of profiles |
| CMI with medial longitudinal arch profile | Arts et al, 2015  Arts et al, 2012^21,22^ | Used as part of custom made feature to modify footwear | Static/dynamic impressions in foam box to individualise profile; modified using pressure data with additional arch support added at times |
| CMI with medial longitudinal arch profile | Paton et al, 2014;  Paton et al, 2012  ^49,50^ | Prefabricated insoles with medial longitudinal arch profile | CMI individualised with prescription protocol for foot deformity; prefabricated profile based on shoe size |
| CMI with medial longitudinal arch profile | Lott et al, 2007  ^45^ | Barefoot, shoe and CMI with metatarsal pad addition conditions | No insole prescription or manufacture reported, no specification of profile |
| CMI with medial longitudinal arch profile | Fernandez et al, 2013^34^ | Used as one component of custom made footwear | Foam box impression with pathology dependent prescription profile - for bony prominences a poron longitudinal inner arch piece also embedded |
| CMI with medial longitudinal arch profile | Reiber et al, 1997;  Reiber et al, 2002^54,55^ | Usual footwear | Used as one component in conjunction with specialist shoe plantar foot scanned to create individual profile; fitted to patient with modifications, no other specification of profile |
| CMI with medial longitudinal arch profile | Rizzo et al, 2012^56^ | Standard treatment | Foam box, static impression to individualise profile but no specifications |
| CMI with medial longitudinal arch profile | Uccioli et al, 1995^61^ | Non-therapeutic shoes | Shaped by cast but no specification of profile |
| Insole containing medial longitudinal arch profile | Scherer 1975^58^ | Used as one component of custom made footwear | Proximally located medial arch within shoe to tip the heel into a varus position, location based on generalisation of foot size |
| CMI with medial longitudinal arch profile | Albert & Rinoie 1994^20^ | Without orthotic | Rigid device from plaster of paris casts, no specification of profile |
| CMI with medial longitudinal arch profile | Burns et al, 2009^25^ | Flat insoles | Use of plaster casts to individualise arch for CMI; no specifications of profile |
| CMI with medial longitudinal arch profile | Bus et al, 2011^26^ | Used as one component of custom made footwear | Moulded base to individualise profile, modifications decided by clinician; no specification of profile or location of rocker |
| CMI with medial longitudinal arch profile | Bus et al, 2004^27^ | Flat insole | Cad-cam, tracings of feet and pressure data to individualise profile; medial longitudinal arch profile plus heel cups (no direct specifications) with other modifications |
| Total contact insole (TCI) with medial longitudinal arch | Hastings et al, 2007^37^ | TCI distal metatarsal pad, TCI proximal metatarsal pad | Foam box impression to individualise profile, no other specifications of profile |
| CMI with medial longitudinal arch profile | Tang et al, 2014^38^ | Prefabricated insoles with medial longitudinal arch | Moulds to individualise to profile, no other specifications of profile |
| CMI with medial longitudinal arch profile | Kastenbauer et al, 1998^41^ | Barefoot, Oxford-style shoe, original cork insole | Customised orthopaedic diabetic insole, no prescription, manufacturing or specifications reported |
| CMI with medial longitudinal arch profile | Mohamed et al, 2004^46^ | Two CMI’s constructed of different materials | No specification of manufacturing process or profile reported |
| Total contact orthoses with medial longitudinal arch | Nouman et al, 2017^66^ | Without total contact insole | Foam box to individualise profile, modified according to a static blueprint, no other specifications |
| CMI with medial longitudinal arch profile | Owings et al, 2008^48^ | Conventionally manufactured CMI’s | Foam box to individualise profile with plantar pressure data |
| CMI with medial longitudinal arch profile | Telfer et al, 2017^68^ | Shape based arch profile | Foam box to individualise profile but manufactured using shape, pressure and ultrasound data |
| CMI with medial longitudinal arch profile | Viswanathan et al, 2004^63^ | No CMI | Positive mould cast to individuals profile, no other specifications |
| CMI with medial longitudinal arch profile | Waajiman et al, 2012^64^ | Used as part of custom made feature to modify footwear | Mould or cast of foot to individualise profile, no other specifications |
| Non-moulded insole | Busch & Chantelau, 2011^28^ | Group not provided with footwear and insole | Flat profile to fit inside therapeutic shoe |
| Unsure | Perry et al, 1995^51^ | Oxford style shoe with no insole | Reported insole inside running shoe but no description of profile |
| Pre-fabricated insole | Barnett 2002^23^ | Flat insole | Non-bespoke standardised specification of arch dependent on shoe size |
| Arched insole | Chantelau et al, 1990^29^ | n/a | Constructed according to shape of the foot and corrected until satisfactory, but no specifications or manufacture technique reported |
| CMI with medial longitudinal arch profile | Ulbrecht et al, 2014^62^ | Standard care orthoses | Foam box and digital scan to individualise profile, with intervention orthoses modified by plantar pressure data |
| CMI with medial longitudinal arch profile | Parker et al, 2019 ^73^ | Flat insoles | Medial arch profile with 10mm heel cup formed by either foam box or weight bearing scan. Weight bearing insoles templates were adjusted by an orthotist, but not disclosed if arch was adjusted |
| Unsure | Soulier 1986^59^ | n/a | Running shoe insole with generic reasonable structure shoe, no specifications of profile |
| Unsure | Lobmann et al, 2001^44^ | No insole | No description or specification of insole profile within shoe |
| CMI with medial longitudinal arch profile | Guldemond et al, 2007^36^ | Different insole configurations including modifications of profile by reducing arch profile by 5mm, adding 5mm and 10mm arch supports | Casted foot to individualise profile |
| Medial longitudinal arched profile | Lin et al, 2013^43^ | Flat insole | Latex arch support, placed under talus, navicular and base of 1^st^ metatarsal, added to insole with double sided tape; size chosen to ensure sub-talar joint neutral position |
| Medial longitudinal arch profile reported as customized insoles | Raspovic et al, 2000^53^ | Without insole | 10 insoles of Non cast type – adhering pieces of D-shaped pad on flat base of medial longitudinal arch area; two neutral shell insoles. No reporting of specifications and positioning of pad. |
| CMI – custom moulded insole, TCO-total contact orthotic | | |  |
