# Supplementary material for: Footwear and insole design features for offloading the diabetic at risk foot—A systematic review and meta‐analyses
Source: Endocrinol Diabetes Metab. 2020 Apr 11;4(1):e00132. doi: 10.1002/edm2.132 (PMC7831212; doi:10.1002/edm2.132)
Supplement: Supplementary file 4 — Appendix S4 [file EDM2-4-e00132-s004.docx]

| Electronic supplementary material 4 rocker profile | | | |
| --- | --- | --- | --- |
| Rocker modification | Studies (n=20) | Comparator | Comments |
| Stiff bottom rocker with early pivot to shoe outsole | Rizzo et al, 2012^56^ | Standard care (no footwear) | Generalised specifications used on participants with previous ulceration or forefoot amputation, marked deformities, hallux amputation and hollow foot with claw toes |
| Urethane (Meramec Group, Sullivan, MO) rocker bottom to shoe outsole – men semi rocker forefoot made rigid with composite shank, women’s shoes semi-rockered with non extended steel shank | Reiber et al, 1997;  Reiber et al, 2002^54,55^ | Usual footwear | No specifications of rocker profile reported; specified treatment objective to generate a smooth rolling motion from heel to toe with normal gait to decrease range of motion in tarso-metatarsal joints and reduce gait induced plantar stress- |
| Semi rigid rocker to shoe outsole | Uccioli et al, 1995 | Non therapeutic shoes | Developed according to Towey guidelines; No further specifications reported |
| Semi rigid rocker to shoe outsole | Wrobel et al, 2014 ^61,65^ | Used in conjunction with different types of insoles | No further specifications reported |
| Anteroposterior rigid rocker to shoe outsole | Lopez-Moral et al, 2019 ^70^ | Semi-rigid rocker sole(Wellwalk technology with Vibram strips) | 20 ° rocker angle between floor and sole under metatarsal heads with rigid (composite fibre) rocker |
| Stiff convex walking sole to diabetic shoe | Busch & Chantelau, 2011^28^ | Group not provided with footwear and insole | No specifications reported but aims to decrease plantar pressure beneath metatarsal heads and prolong pain free walking |
| EVA micro rubber sole on therapeutic footwear | Paton et al, 2014;  Paton et al, 2012  ^49,50^ | Used in both intervention and control groups | Rocker added to forefoot positioned posterior to the metatarsal phalangeal joint line |
| Rigid rocker constructed of 1/16 x 1-inch spring steel shank embedded under the outsole of shoe | Owings et al, 2008^48^ | Flexible shoe | Rocker angle 20°, located at 65% of the sole length as measured from the heel |
| Rocker to outer sole of shoe | Chapman et al, 2013^30^ | 12 different rocker designs | 12 variations in apex angle (relative to metatarsal break), apex position (normalised to shoe length), rocker angle |
| EVA and 5mm folex rocker addition to the outsole of a standard shoe (Duna, Italy) | Preece et al, 2017^67^ | Eight different rocker designs | Eight variations in rocker angle (15° or 20°) and apex position (52, 57, 62 and 67% from the rearfoot) |
| Either 1cm forefoot rocker (excessive pressure under 1^st^ and- 5^th^ MTPJ) or 1cm ‘u’ shaped rocker (excessive pressure under central metatarsals) to external sole of shoe | Fernandez et al, 2013^34^ | Used as one component of custom made footwear | Rocker feature prescribed when increased vertical pressure in push-off stage of walking gait (hallux rigidus, functional limitus, 1^st^ ray amputation or digit amputation) assessed by barefoot plantar pressure platform analysis. |
| Diabetic footwear with rocker outer soles | Hsi et al, 2004 ^40^ | Patients own shoes | Rocker sole addition comprised of 11mm height, 29mm thickness at the heel, 16mm at the front end and 24mm at the maximum of the rocker curve. The rocker started to curve up 83mm from the front end at the medial side and 87mm at the lateral side |
| Anterior wedge rocker added to insole | Frykberg et al, ^35^ | Surgical boot without insole, patients’ own Oxford or tennis style shoes | Rocker modification of dense closed cell foam applied to the insole proximal to the metatarsal heads contained within a surgical boot |
| Rubber made walking sole shaped to rocker | Chantelau et al, 1990^29^ | n/a | No specifications of rocker |
| Stiffened rubber outsole and roller configuration to shoe | Bus et al, 2011^26^ | Used as one component of custom made footwear | Pressure informed modification of adding earlier or more significant rocker or roller either in shoe or outside shoe |
| Semi-rigid outer sole or stiff rocker bottom | Tang et al, 2014^38^ | Used in both intervention and control group | No specifications reported |
| Longitudinal outsole curvature | Praet et al, 2003 ^52^ | Variations in shoe and insole modalities | Variations of rocking axis position (60%, 61.5%, 63%, 65%, 67.5%) and rocking angle (5°, 8°, 10°, 23°) |
| Stiffened rubber outsole and roller configuration to shoe | Waajiman et al, 2012^64^ | Used as one component of custom made footwear | Generalised construction with no specifications reported |
| Legend: MTPJ – Metatarsal phalangeal joint, EVA-Ethylene-vinyl acetate, n/a not applicable | | | |
