# Supplementary material for: Footwear and insole design features for offloading the diabetic at risk foot—A systematic review and meta‐analyses
Source: Endocrinol Diabetes Metab. 2020 Apr 11;4(1):e00132. doi: 10.1002/edm2.132 (PMC7831212; doi:10.1002/edm2.132)
Supplement: Supplementary file 5 — Appendix S5 [file EDM2-4-e00132-s005.docx]

| Electronic supplementary material 5 - modifications to footwear | | | |
| --- | --- | --- | --- |
| Extra depth modification | Studies (n=35) | Comparator | Comments |
| **Off the shelf footwear** | | |  |
| Diabetic footwear (County Orthopaedic Footwear Ltd, UK) | Paton et al, 2014;  Paton et al, 2012  ^49,50^ | Used in both intervention and control groups | Standardised footwear with more depth and width |
| Extra depth or DX2 footwear (p.w. Minor, Batavia, NY). | Ulbrecht et al, 2014^62^ | Used in both intervention and control groups | Standardised footwear but could be adjusted at fitting to include stretching |
| Extra depth footwear (Dr Comfort, DJO, UK) | Wrobel et al, 2014 ^65^ | Used in both intervention and control groups | Standardised off-the-shelf-footwear |
| Extra depth footwear Sir Super Depth (p.w. Minor, Batavia, NY) 55 Durometer, 18 iron. | Albert & Rinoie 1994^20^ | Used in both intervention and control groups | Not disclosed if patient specific |
| Extra depth | Rizzo et al., 2012^56^ | Standard care | Semi-orthopaedic footwear on market with extra depth to fit Custom made insoles. Not clear if patient specific. |
| Extra width, depth and height (DVA/Seattle Footwear, US). | Reiber et al., 1997;  Reiber et al., 2002^54,55^ | Used in both intervention and control groups | Prototype footwear Extra width and height to toe box, increased depth to length of shoe. Not clear if patient specific. |
| Extra depth, width and height(Podartis s.r.l. Unipersonale – Crocceta del Montello, Italy) | Lopez-Moral et al, 2019 ^70^ | Used in both intervention and control groups | Therapeutic shoes with high toe box, enough width to accommodate toe deformities, depth 14 or 16mm deeper than standard footwear. |
| Extra depth (Thermomold, NY) | Birke et al, 1999^24^ | Used in all interventions | Standardised off-shelf-shoe; not patient specific |
| Extra depth(Sole Tech, Advanced Orthopedic Footwear, style number E3010) | Hastings et al, 2007^37^ | With and without insoles | Advanced orthopaedic footwear prescribed according to shoe size |
| Extra depth (Finn Comfort, Germany). | Kastenbauer et al, 1998^41^ | Oxford style shoes | Standardised shoe. Unsure if patient specific |
| Standard diabetic shoes (Dr. Foot Technology Co, Taiwan) | Lin et al, 2013^43^ | Used in all interventions | Xtra depth leather shoes |
| Extra depth | Telfer et al, 2017^68^ | Used in all interventions | Only prescribed for use in trial runs |
| Suitable depth | Raspovic et al, 2000^53^ | Used in all interventions | Footwear modified to be of ‘suitable’ depth, but no specifications reported. |
| Extra deep diabetic shoes (Dr Kong Footcare Ltd. Taiwan) | Tsung et al, 2004^60^ | Used by all participants | Shoe selected to size, according to Tovey’s principles. The first metatarsophalangeal joint should be accommodated in the widest part of the shoe and the length should allow 1-1.25cm between the end of the shoe and the longest toe |
| **Bespoke footwear** | | |  |
| Ready-made diabetic footwear (Orthoaktiv, F.W. Kraemer, Remscheid, Germany) | Hsi et al, 2002^39^ | Patients’ own shoes | Standardised diabetic footwear |
| Extra depth or fully customised footwear | Arts et al, 2015  Arts et al, 2012^21,22^ | Used by all participants | Either ‘Extra-depth’ off-the-shelf footwear or custom footwear made from last derived plaster cast of foot |
| Extra depth footwear | Fernandez et al, 2013^34^ | Used by all participants | Prescribed footwear according to length and width of foot, using Dahmen’s algorithm. |
| Extra depth shoes | Uccioli et al, 1995^61^ | Ordinary shoes | Footwear designed according to Towey guidelines with super depth to fit insoles and toe deformities. Not clear if patient specific. |
| Extra width and depth | Scherer 1975^58^ | Used by all participants | Manufactured according to shoe-size, foot width and length. Bespoke to patient. |
| Extra depth protective shoes(Thanner, Germany) with deep soft uppers and no toe-caps with a firm heel counter | Lobmann et al, 2001^44^ | Used by all participants | Protective shoe manufactured according to Tovey’s model. Unsure if patient specific |
| Customised footwear or extra depth | Bus et al, 2011^26^ | Used by all participants | Participants received either ‘Extra-depth’ off-the-shelf footwear or custom footwear made from last derived plaster cast of foot |
| Customised diabetic footwear | Praet & Louwerens 2003^52^ | Standardised footwear: rubber soled Oxford style shoe (model 7143-A, Vab der Hammen B.V. Waalwijk, the Netherlands), Xtra depth Oxford shoe (model 3116, Bimakon Hederland BV, Drunen, NL), Xtra-depth Diabetic shoe (Nimco Orthropedics, Berg en Dal, the Netherlands),  Xtra-stretched shoe Nimco Orthropedics, Berg en Dal, the Netherlands) | Shoes fabricated by orthotist |
| **Retail footwear** | | |  |
| Running shoes (New Balance trainers 460, US) with accommodative padding added into insole, width sizing and smooth outsole pattern to reduce tripping indoors | Soulier 1986^59^ | Used by all participants | Retail-footwear not patient specific |
| Extra width and depth running shoes (SAS, San Antonio, TX, US or New Balance, Boston, MA, US), | Donaghue et al, 1996^33^ | Used by all participants | Retail footwear not patient specific |
