# Supplementary material for: Footwear and insole design features for offloading the diabetic at risk foot—A systematic review and meta‐analyses
Source: Endocrinol Diabetes Metab. 2020 Apr 11;4(1):e00132. doi: 10.1002/edm2.132 (PMC7831212; doi:10.1002/edm2.132)
Supplement: Supplementary file 6 — Appendix S6 [file EDM2-4-e00132-s006.docx]

| Electronic supplementary material 6 - metatarsal modifications | | | |
| --- | --- | --- | --- |
| Metatarsal modification | Studies (n=18) | comparator | Comments |
| Metatarsal pad or metatarsal bar | Bus et al, 2011 Bus et al, 2004 ^26,27^ | n/a | Option of use being incorporated into Total Contact Insole chosen by orthopaedic shoe-maker to reduce Peak Pressure in Regions Of Interest based on PP data, tracings and static footprints in conjunction with other modifications |
| Metatarsal pad or metatarsal bar | Arts et al, 2015  Arts et al, 2012  ^21,22^ | n/a | No clear description of position, size, material, shape of pad or bar. Chosen as modifications by shoe technicians and repositioned to reduce PP in ROI >200kPa. Used in conjunction with arch support on occasion |
| Metatarsal pad and metatarsal bars | Ulbrecht et al, 2014^62^ | n/a | No clear description of position, size, material, shape of pad or bar. Option of being incorporated into insole prescription for sub-metatarsal offloading Decision to use based on opinion of orthotist |
| Metatarsal pad | Hastings et al, 2007; Lott et al, 2007; Mueller et al, 2006 ^37,45,47^ | Three sizes of metatarsal pad made of cork, shore value 55°, selected to cover three central metatarsal heads, | Metatarsal pad applied to Total contact insole with adhesive backing. Orthotist/pedorthotist drew line to determine metatarsal head location for placement 1cm proximal. |
| Pre-metatarsal bar | Rizzo et al, 2012^56^ | n/a | No clear description of position, size, material or shape of bar. Used in conjunction with a medial arch support. Used based on an individualised strategy based on consensus of three clinicians to lower high forefoot pressures. |
| Metatarsal pads 2^nd^ to 4^th^ MTPJ | Mohamed et al, 2004 ^46^ | n/a | No clear description of size, material or shape of bar. Added to six of 16 insoles after one month of use due to excessive wear or bottoming out in opinion of orthotist. |
| Metatarsal bar | Tang et al, 2014^38^ | n/a | No clear description of material or shape of bar. Standardised bar, fitted proximal to the 2^nd^ to 4^th^ metatarsal heads within the CMI and prefabricated insoles. Adjustments including raising or lowering bar height, but no specifications or rationale given. |
| Metatarsal bar | Owings et al, 2008^48^ | n/a | No clear description of size, material or shape of bar. Created within Total Contact Insole from automated design algorithm which identified pressure contour of MTPJ’s. |
| Metatarsal dome | Guldemond et al, 2007^36^ | n/a | 11mm high foam rubber (Shore A 28) dome, positioned 5mm behind the 2nd to 4^th^ metatarsal heads on the insole. Positioned from dynamic pressure sheet footprint. |
| Metatarsal or central head mounds | Fernandez et al, 2013^34^ | n/a | No clear description of size, material or shape of bar. Used when elevated pressure over static bony prominence when joints were mobile in forefoot zone. |
| Metatarsal bars or pads | Telfer et al, 2017^68^ | n/a | No clear description of position, size, material or shape of bar or pads. Manufacturer could use this if felt appropriate as per standard practice for CMI; met bar increased in height to reduce peak pressure in cad cam design |
| Metatarsal pad or bar | Parker et al, 2019 ^73^ | n/a | Used on two of the insoles at discretion of orthotist based on static pressure footprints. No clear description of position, size, material or shape of bar or pads |
| Metatarsal bar | Martinez-Santos et al, 2019 ^71^ | n/a | Distal location and shape defined where plantar pressure was 77% of the peak pressure. Used in combination with different void conditions |
| Metatarsal aperture | Barnett 2002^23^ | n/a | Located at widest part of forefoot with material removed from insole and replaced by softer material into insole at metatarsal |
| PP=peak pressure; ROI=regions of interest; MTPJ=metatarsal phalangeal joint; n/a not applicable | | | |
