# Supplementary material for: Footwear and insole design features for offloading the diabetic at risk foot—A systematic review and meta‐analyses
Source: Endocrinol Diabetes Metab. 2020 Apr 11;4(1):e00132. doi: 10.1002/edm2.132 (PMC7831212; doi:10.1002/edm2.132)
Supplement: Supplementary file 7 — Appendix S7 [file EDM2-4-e00132-s007.docx]

| Electronic supplementary material 7 - cut outs or aperture modifications | | | |
| --- | --- | --- | --- |
| Feature | Studies (n=12) | Comparator | Comments |
| Removal of material | Arts et al, 2015  Arts et al, 2012  ^21,22^ | n/a | Removal of material at high pressure areas identified by pressure data, tracings and static blueprint |
| Removal of material | Bus et al, 2011^26^ | n/a | Removal of material to reduce peak pressure at regions of interest identified by in-shoe system |
| Removal of material | Bus et al, 2004^27^ | n/a | Removal of material at areas of high pressure identified by pressure data, tracings and static footprint |
| Removal of material | Waajiman et al, 2012^64^ | n/a | 33% of insoles modified by removal of material at ROI identified by PP in-shoe system |
| Cut out | Lopez-Moral et al, 2019 ^70^ | n/a | Cut out positioned at the previously ulcerated metatarsal head |
| Fenestrations | Fernandez et al, 2013^34^ | n/a | 6mm poron plug embedded in fenestrations for areas of high pressure and bony prominence and joints which showed insufficient mobility for selective offloading |
| Removable square plugs | Lin et al, 2013^43^ | Pre-plug removal | Plugs 1cm x 1cm removed in forefoot area for ROI (highest mean peak pressure) |
| Aperture | Owings et al, 2008 ^48^ | n/a | 3mm deep aperture for regions of excessive pressure >1000kPa |
| Aperture or u shaped rubber | Raspovic et al, 2000^53^ | n/a | Sited under previous ulcerated site |
| Removal of material under metatarsal head | Telfer et al, 2017^68^ | n/a | Used to reduce regional MPP to under 200kPa informed by finite element modelling |
| Local removal of material or softening of material | Parker et al, 2019 ^73^ | n/a | Utilised on seven of the insoles at discretion of orthotist, informed by static pressure footprints. |
| 3mm void conditions | Martinez-Santos et al, 2019 ^71^ | Different void conditions created with altering material (no material, poron (20 Shore A), EVA (20 Shore A) alongside different metatarsal bar combinations | Distal border of void placed distal to area of peak pressure and used in conjunction with metatarsal bar; |

n/a not applicable, ROI Region of Interest
