# Supplementary material for: Footwear and insole design features for offloading the diabetic at risk foot—A systematic review and meta‐analyses
Source: Endocrinol Diabetes Metab. 2020 Apr 11;4(1):e00132. doi: 10.1002/edm2.132 (PMC7831212; doi:10.1002/edm2.132)
Supplement: Supplementary file 8 — Appendix S8 [file EDM2-4-e00132-s008.docx]

| Electronic supplementary material 8 - Casting technique | | | |
| --- | --- | --- | --- |
| Casting technique | Studies n=23 | Comparator | Comments |
| Plaster of Paris | Albert & Rinoie 1994^20^ | n/a | No technique disclosed |
| Plaster of Paris | Burns et al, 2009^25^ | n/a | Neutral suspension technique |
| Plaster of Paris | Tang et al, 2014^38^ | n/a | Positive mould based on negative cast; patient prone positioned |
| Plaster of Paris or foam box | Arts et al, 2015  Arts et al, 2012^21,22^ | n/a | Positive cast with additional modifications informed by shoe technician |
| Plaster of Paris | Viswanathan et al, 2004^63^ | n/a | Positive mould, no other specifications |
| Plaster of Paris | Coagiuri et al, 1995^31^ | n/a | STJ neutral, mid-tarsal maximally pronated. |
| Plaster of Paris or foam box | Waajiman et al, 2012^64^ | n/a | No technique disclosed |
| Foam box | Rizzo et al, 2012^56^ | n/a | Feet in neutral, knees 90°. Used with information from static footprint |
| Foam box | Nouman et al, 2017^66^ | n/a | Sub talar joint in neutral, knees 90°. Modifications informed by information from static footprint |
| Foam box | Paton et al, 2014;  Paton et al, 2012  ^49,50^ | n/a | Cad-Cam technique to mill Custom Made Insole |
| Foam box | Hastings et al, 2007^37^ | n/a | Design and modifications based on clinical decision by orthotists |
| Foam box | Owings et al, 2008^48^ | n/a | No technique disclosed |
| Foam box | Lott et al, 2007^45,47^ | n/a | No technique disclosed |
| Foam box | Nouman et al, 2019 ^72^ | n/a | Cast obtained by a qualified orthotist; no other specifications disclosed |
| Foam box | Tsung et al, 2004^60^ | Fully weight-bearing (standing on casting foot only) compared with semi-weight-bearing (standing only) with non-weight-bearing (sitting, ankle neutral, knee 90°) |  |
| Cad-cam | Bus et al, 2011 Bus et al, 2004^26,27^ | n/a | Based on plantar pressure data, tracings and footprint |
| Laser digitizer | Reiber et al, 1997;  Reiber et al, 2002^54,55^ | Standard preformed polyurethane insole | Weight-bearing, static image of contours of foot uploaded into software which creates 3D image of foot |
| Digital AMFIT (AMFIT Incorporated, Vancouver, WA, USA) system | Wrobel et al, 2014 ^65^ | Standard insoles | Image of foot digitized and used to manufacture insoles and Dynamic Foot Orthoses |
| ‘Cast’ | Uccioli et al, 1995^61^ | n/a | No technique disclosed |
| Foam box, cad cam, finite element | Telfer et al, 2017^68^ | Shape date and milling produced insole | Individualised for each patient with different techniques compared to inform manufacturing processes |
| Foam box and weightbearing digital foot scan | Parker et al, 2019 ^73^ | Flat 3mm poron insole | Foam box devices manufactured with plaster impression, heat moulded to cast and hand finished by blinded technicians. Digital scan from barefoot standing and modified by orthotist based on static pressure data. |

n/a not applicable
