# Supplementary material for: Footwear and insole design features for offloading the diabetic at risk foot—A systematic review and meta‐analyses
Source: Endocrinol Diabetes Metab. 2020 Apr 11;4(1):e00132. doi: 10.1002/edm2.132 (PMC7831212; doi:10.1002/edm2.132)
Supplement: Supplementary file 9 — Appendix S9 [file EDM2-4-e00132-s009.docx]

| Electronic supplementary material 9 - fabrication informed by kinetic parameters | | | |
| --- | --- | --- | --- |
|  | Studies (n=11) | Comparator | Comments |
| Pedar-X (Novel, GmbH, Munich, Germany) in-shoe system | Bus et al, 2011; Waajiman et al, 2012; Waajiman et al, 2012 ^21,26,64^ | Used for all participants | Identify regions of interest>200kPa in the midfoot or forefoot; modified insoles using a set algorithm with up to three rounds of modifications to achieve regions of interest optimisation (25% below MPP or <200kPa). |
| Pressure platform (Novel EMED-SF, USA) for barefoot pressures | Bus et al, 2004^27^ | Standard insole | Used barefoot plantar pressure data to inform custom made insole |
| Pedar X (Novel, GmbH, Munich, Germany) in-shoe system | Lin et al, 2013^43^ | Used for all participants | Plugs were removed from the insole at the Region of interest=highest MPP |
| RScan (RScan International Lammerdries, Belgium) platform for barefoot | Fernandez et al, 2013^34^ | Used for all participants | Used barefoot plantar pressure data and radiophotopodogram findings to inform for selective offloading using insoles |
| Static footprint taken with the patient standing barefoot | Rizzo et al, 2012^56^ | Standard treatment | Used in conjunction with foam box impression of feet to identify problem areas requiring attention by three professionals in discussion. |
| Pedar (Novel, GmbH, Munich, Germany) in-shoe system for in-shoe plantar pressures | Reiber et al, 1997^54^ | Standard insole | Data is used to create a 3D image template, from which the custom insole is milled from cork blanks, with modifications identified by physical landmarks, foot exam and foot pathology |
| EMED (Novel, GmbH, Munich, Germany) platform for barefoot dynamic testing | Owings et al, 2008 ^48^ | Insoles not designed by pressure data | Data used in conjunction with foam box cast and computer display to create insole with metatarsal bar and 3mm deep area aperture in areas >1000kPa; |
| Dynamic pressure sheet footprint to determine the locations of the metatarsals to position the metatarsal domes. | Guldemond et al, 2007 ^36^ | n/a | Used in conjunction with foam box casting |
| Static pressure collected with platform (Emed platform, Novel, GmbH, Munich, Germany) | Martinez-Santos et al, 2019 ^71^ | Used for all participants | Used in conjunction with 3D foot shape captured by scanner (Inescop, Spain) |

n/a not applicable
