# Supplementary material for: Footwear and insole design features for offloading the diabetic at risk foot—A systematic review and meta‐analyses
Source: Endocrinol Diabetes Metab. 2020 Apr 11;4(1):e00132. doi: 10.1002/edm2.132 (PMC7831212; doi:10.1002/edm2.132)
Supplement: Supplementary file 10 — Appendix S10 [file EDM2-4-e00132-s010.docx]

| Electronic supplementary material 10 – materials of insole and footwear | | | |
| --- | --- | --- | --- |
| Materials | Studies (n=37) | Comparator | Comments |
| TL-2100 graphite with Naugahyde top cover (P.W. Minor and Sons, Batavia, NY) | Albert & Rinoie 1994^20^ | n/a | Dual density and rigid device aimed at placing abnormal foot in an optimal functioning position; used only in pronated foot posture participants |
| 10mm thick rubber-foam (Zellkautschuk, Berkemann, Hamburg, Federal Republic Germany) and other plastics (PPT and Platazote; Schein, Remscheid, Federal Republic Germany) insole; soft leather shoe | Chantelau et al, 1990^29^ | n/a | Dual density insole designated ‘cushioned’; thickness thought to attenuate greater force reduction |
| Shoes made from soft leather upper, outersole of microcellular rubber, with 5mm folex for rocker (Duna, Falconara Marittima, Italy) | Chapman et al, 2013^30^ | n/a | Materials selected to prevent flexion of shoe |
| Rohadur thermal plastic (Ozthotics, Randwick, NSW, Australia | Colagiuri et al, 1995^31^ | n/a | Material choice for ‘control’ of foot function to reduce plantar foot pressures |
| Thor Lo hosiery (Thor-Lo, Statesville, NC) | Donaghue et al, 1996 ^33^ | n/a | Unknown materials of shoes or hosiery |
| For static pressures: heat moulded laminar EVA insole (25°-60° Shore) base with a 3mm PPT layer  heat moulded laminar EVA (25°-60° Shore) For pressure and boney prominence: EVA insole (25° -33° Shore) with maximum thickness of 1.5-2cms with high density EVA (60° Shore) bottom layer. 6mm Poron used to offload specific areas | Fernandez et al, 2013^34^ | n/a | Different density materials used dependent on whether pressure static or pressure coincided with bony prominence. Shock absorbing material used in areas of bone protrusion or previous ulcer and wound sites. |
| Multi-layer orthosis EVA orthosis (40°Shore) with poron top cover; shoe material made of soft skin | Lopez-Moral et al, 2019 ^70^ | n/a | No rationale for material choice |
| Alipast and plastazote (Voltek, Brebbia, VA) insoles | Mohamed et al, 2004^46^ | Plastazote (Zotefoams Inc., Walton, KY) | Combination used to theoretically increase longevity of insoles |
| Insole 1:thin polypropylene shell with Korex, sponge or plasatazote top cover; Insole 2: 45 Shore S EVA base with Procell or plastazote top cover; Insole 3: 35 Shore A Microcel Puff EVA base and a Poron or P-Cell top cover | Owings et al, 2008^48^ | n/a | Dual density insoles with materials selected as commonly used in offloading. |
| 3mm medium density EVA base with 6mm Poron top cover | Paton et al, 2014;  Paton et al, 2012  ^49,50^ | n/a | Dual density for both the prefabricated and customised insole aimed at reducing plantar pressure; durability also measured after 12 months |
| Medium density rubber cork inserts, 1.5mm layer foam backed nylon tricot top layer  Shoes made of high quality cowhide leather with urethane (Meramec Group, Sullivan, MO) outersole | Reiber et al, 1997;  Reiber et al, 2002^54,55^ | Standard study insole: closed cell polyurethane foam | Dual density insole; cork used for little set or deformation and top cover aims for ‘cushioning interface between foot and insole |
| Shoes made of Bottine, soft thermformable leather; insoles made of PPT (Deer Park NY), Duoterm (Mibor, Alcoy, Spain) and Alcaform (Zotefoams Plc, Croydon, UK) | Rizzo et al, 2012^56^ | n/a | PPT to relieve local pressure, Duoterm and Alcaform to absorb high pressure points |
| Natural leather skin upper, synthetic rubber sole | Scherer 1975^58^ | n/a | No rationale provided |
| Shoes made of soft thermformable leather; Insoles made of Alcapy (Deer Park, NY) and Alcaform | Uccioli et al, 1995^61^ | n/a | Alcapy to relieve local high pressures and Alcaform to absorb high pressure points |
| 8mm Polylux, 8mm Combilux, 2.3mm Memorix, 3mm Remember and 0.7mm Calbino topcover (Thanner, GmgH, Hochstadt, Germany) | Burns et al, 2009^25^ | Flat 4mm EVA and 0.7mm Calbino topcover (Thanner, GmgH, Hochstadt, Germany) | Mesh of materials combined; no rationale provided |
| Diabetiker SY2 modular viscoelastic insole of 2.5mm polyvinyl chloride (Kraemer, Remscheid, Germany) | Hsi & Lai, 2002; Hsi et al, 2004^39,40^ | n/a | to act as shock absorbers with24 sensors embedded in insole |
| Shoe made from EVA and rubber (Softgummi) sole, cloth, rubber foam and leather uppers Insole made of: Rear part containing 6mm lunasoft, 42° Shore A hardness; anterior part 6mm Lunaflex, 20° Shore A hardness; covered with 3mm thick PPT, 17° Shore A hardness) | Busch & Chantelau, 2011^28^ | n/a | Soft density upper to avoid toe pressure strain, firmer density rocker sole to decrease plantar pressures beneath metatarsal heads and prolong pain free walking; Tri-density, non-moulded insole aimed at cushioning forefoot area. |
| 5mm Lunalastick and 8mm Lunasoft SL (NORA, Freudenberg, GmbH, Weinheim, Germany) top and bottom and 1.1mm Rhenoflex 3208 (Rhenoflex, GmbH, Ludwigshafen, Germany) | Guldemond et al, 2007^36^ | n/a | Higher stiffness materials above Shore A 60° used to minimize the influence of cushioning on plantar loading |
| 3mm Shore A 35° EVA in the first layer, 2mm Velcro and velvet in the second layer and 6mm Shore A 50° Poron in the third layer | Lin et al, 2013^43^ | n/a | No rationale for material choice |
| 14mm multi-combination insole EVA, polyethylene foam, elastomere, silicone | Lobmann et al, 2001^44^ | n/a | Silicone with special arrangement to achieve the required degree of hardness |
| Custom made insole open cell urethane foam hardness 60-80 (Langer, Inc, Deer Park, NY, USA) with the addition of 2mm base and 0.7mm top cover | Bus et al, 2004^27^ | Flat insole 0.95cm thick PPT(Langer, Inc, Deer Park, NY, USA) | Dual density materials frequently prescribed in diabetic foot practice |
| Multifoam as the top layer, Plastazote (Streifeneder ortho production GmbH, Emmering, Germany) as the second layer and microcellular rubber as the final stabilising layer | Nouman et al, 2017^66^ | n/a | No rationale for materials given |
| 5mm thick multifoam (30° Shore A hardness), 8mm thick Plastazote (25° Shore A Hardness) and 10mm thick microcellular rubber (70° Shore A hardness) | Nouman et al, 2019 ^72^ | Dual density insole of 8mm thick Plastazote (25° Shore A Hardness) and 10mm thick microcellular rubber (70° Shore A hardness) | Hypothesised that different combinations of materials would influence peak pressure and contact area |
| Rohadur (Ozthotics, Randwick, NSW, Australia) device with dual acrylic posts added to rearfoot to balance foot | Coagiuri et al, 1995^31^ | n/a | Rigid orthotic to provide functional control providing foot contact shock absorption phase during normal pronation, midtarsal stability and propulsive thrust |
| Insole made of closed-cell polyurethane foam and soft insole cover | Frykberg et al, 2013^35^ | n/a | No rationale provided |
| Dynamprene (neoprene based, Dupont) built into shoe sole of trainer; | Kastenbauer et al, 1998^41^ | Barefoot, cork insole multilayer insole and in-depth custom insole made up of 10 different layers (Schein Orthopadie Service, Reinscheid, Germany) | Aimed at shock-absorbing but not specific to patient |
| 1.27cm #2 plastizote (Shore 35°), 5.0mm thick cross-linked polyethelene foam blended with EVA insole and Cork (Shore 55°) met-pad | Hastings et al, 2007; Lott et al, 2007; Mueller et al, 2006 ^37,45,47^ | n/a | No rationale provided |
| ¼” thick Poron 14°Shore Hardness | Birke et al, 1999^24^ | Seven (17°,22°, 27°, 32°, 40°, 50° Shore hardness) densities of Poron tested in reducing mean peak pressure | Material selected as most popular non moulded orthosis material to reduce pressure |
| Insole made of Poron 96 (Rogers Corporation, Woodstock, CT) | Cumming & Bayliff 2011^32^ | Insole made of Poron 4400 (Rogers Corporation, Woodstock, CT | One left and one right insole of each material issued to participants; mean total pressure measured after one week duration |
| 35 durometer EVA base and added two non-stick sheets, held with elastic binders, between the upper pad and lower pad of 3mm thick 45 durometer EVA. To this a 3mm thick 20 durometer polyethylene foam top was added | Lavery et al, 2012^42^ | Standard insole made of 35 durometer EVA base, lower pad of 3mm thick 45 durometer EVA. To this a 3mm thick 20 durometer polyethylene foam top was added | Intervention insole aimed at shear and pressure reduction characteristics |
| 4mm cushioned properties | Perry et al, 1995^51^ | n/a | Insole within Nike Air Craft running shoe; no description of materials |
| Padded insoles | Soulier 1986 ^59^ | n/a | Insole within New Balance 460 running shoe; no description of materials |
| Insole made of polyurethane, EVA, or 10mm microcellular rubber insole and 8mm rubber sole, 5mm polyurethane foam insole, 5mm MCR midsole and 10mm EVA outer sole or10mm EVA as outer sole, 6mm cork as midsole and 6mm polyurethane | Viswanathan et al, 2004^63^ | Insole of hard leather board, | Materials selected due to being lightweight, shock absorbent, flexible and highly durable |
| Insoles made of Rubbatex neoprene rubber top cover with 4-way stretch darlex (Richardson Products Incorporated, Frankfort, IL, USA), silicone layer that was based on firm density EVA base lined with ballistic nylon | Wrobel et al, 2014 ^65^ | Standard Insoles made of firm density plastazote and PPT bi-lam (American Plastics Arlington, TX, USA) | Intervention materials selected to decrease compressive forces and reduce sliding friction |
| Custom made insole of Nora Lunasoft A50° hardness (Freudenberg, Germany) and 3mm Poron top cover 3mm thickness | Tsung et al, 2004^60^ | Flat insole made of Nora Lunasoft A50° hardness (Freudenberg, Germany) and 3mm Poron top cover 3mm thickness | No rationale for material choice provided |
| Shoes mainly of leather with rubber outsole; insole of Mouldable cork or multifoam base, open or closed cell material top cover | Bus et al, 2011; Waajiman et al, 2012^26,64^ | n/a | Materials selected as they are commonly used in practice |
| EVA (A35°) with laminated fabric PPT top cover | Ulbrecht et al, 2014^62^ | n/a | No rationale provided |
| Shoes made of stiffened rubber and/or polyethylene reinforced outer sole with insole of Rhenoflex thermoplastic (Ludwigshafen-am-Rhein, Germany) with multifoam or cork base finished with plastazote (Zotefoams plc, Croydon, UK), leather or PPT (Langer Inc, Deer pArk, Ny, USA) top cover | Arts et al, 2015  Arts et al, 2012^21,22^ | n/a | Materials selected using own companies design and manufacturing standards |
| Insoles made of EVA Shore hardness 35° or 55°) | Tang et al, 2014^38^ | Prefabricated insole of mixture of thermoplastic, polyurethane, polyester and polycarbonate | Materials selected to assess ability to reduce kinetic variables |
| Rubber pad on unknown base for most of insoles; one insole of polyproprolene shell and one insole EVA shell | Raspovic et al, 2000^53^ | n/a | No rationale provided |
| EVA insole with 3mm PPT cover; rubber sole leather Oxford shoe; | Praet & Louwerens 2003^52^ | PU-soled Xsensible Xflex shoe; Polyurethane soled shoe; soft leather shoe with insole made of 10mm EVA with 3mm PPT top cover & 3mm rocker; soft leather shoe with insole made of 10mm EVA with 3mm PPT top cover & 3mm rocker | Different shoe and insole material combinations; commonly used materials |
| 6mm Medium density EVA rearfoot (30-40 Shore A), 6mm poron (20 Shore A) at forefoot with topcover of leather | Parker et al, 2019 ^73^ | 3mm flat Poron insole | No rationale provided |
| Insole made of medium density EVA (50° Shore A) with variety of modifications using void conditions (EVA 20° Shore A, Poron 20° Shore A) and | Martinez-Santos et al, 2019 ^71^ | Flat insole made of 3mm EVA 50° Shore A | No rationale provided |
| Prefabricated insole (10mm EVA base Shore A35, upper layer 6mm EVA Shore A 25) EVA and 1mm EVA shore A 25 top cover | Barnett 2002^23^ | Cleron (control insole) | Modifications of heel and metatarsal with non-cellular polyurethane elastomer incorporated into shell of insole |
| Legend: EVA-Ethyl-Vinyl Acetate, PPT – Professional Protective Technology, n/a not applicable | | | |
| Materials | Studies (n=37) | Comparator | Comments |
| TL-2100 graphite with Naugahyde top cover (P.W. Minor and Sons, Batavia, NY) | Albert & Rinoie 1994^20^ | n/a | Dual density and rigid device aimed at placing abnormal foot in an optimal functioning position; used only in pronated foot posture participants |
